# Reversing fertility decline in Japan with foreign pro-natalist policies, 1990–2035: a systematic review and secondary data analysis

**DOI:** 10.1016/j.lanwpc.2025.101596

**Published:** 2025-06-14

**Authors:** Md Mizanur Rahman, Haruka Sakamoto, Sabera Sultana, Miho Sassa, Md Ashraful Alam, Kenji Shibuya

**Affiliations:** a Research Center for Health Policy and Economics, Hitotsubashi Institute for Advanced Study, Hitotsubashi University, Tokyo, Japan; b Tokyo Foundation for Policy Research, Tokyo, Japan; c Department of International Health and Tropical Diseases, Tokyo Women’s Medical University, Japan; d Department of Global Health Policy, Graduate School of Medicine, The University of Tokyo, Japan; e Department of Computational Diagnostic Radiology and Preventive Medicine, The University of Tokyo Hospital, Japan; f Medical Excellence JAPAN, Tokyo, 103-0023, Japan

**Keywords:** Total fertility rate, Birth rate, Fertility policy, Cash incentive, Parental leave, Scenario-based analysis, Japan

## Abstract

**Background:**

Several high-income countries, including Japan, have adopted pro-natalist policy to counteract declining fertility rates. However, the effectiveness of these policies varied across different studies and countries. This study aims to conduct an initial systematic review to identify effective policies that have reversed declining fertility rates. Subsequently, it will apply them to Japan’s context through scenario-based secondary analysis. The goals are to project Japan’s total fertility rate (TFR) up to the year 2035.

**Methods:**

We conducted searches in major electronic databases from their inception until March 27 2025 to investigate the impact of fertility policies. We also extracted a variety demographic and public expenditure data from the OECD, World Bank, and IMF databases for the years 1990–2022. A Bayesian hierarchical regression model was used to estimate and project Japan’s TFR trends up to 2035 in Japan, analyzing the effects of significant fertility policies on these trends.

**Findings:**

Our analysis included a total of 61 studies focusing on pro-natalist policies, identifying cash benefit policies—such as payment at birth, allowances, paid maternity and paternal leave, childcare coverage, and tax exemptions—as the most influential. Unpaid maternity leaves, universal two-child policy, and Assisted Reproductive Technology health insurance coverage also showed potential to boost fertility, although further research is needed. Countries like Australia, Greece, Hungary, France, and the UK allocate over 1.3% of their GDP to family cash benefits, contrasting Japan and Korea, where the allocation is less than 1%. Our findings indicate that Japan’s current cash benefit policies are unlikely to significantly reverse the fertility decline by 2030 (12% likelihood) and 2035 (29% likelihood). However, should Japan enhance its cash benefits to levels observed in Australia, Hungary, and France by 2030, the probability of reversing Japan’s fertility decline could rise significantly to 79%, 70%, and 69%, respectively. Even if cash benefit policies are scaled up by 2035, the probability of reversing the decline fertility rate is projected to be around 60 or more.

**Interpretation:**

Cash benefits emerged as the most effective fertility policy in high-income countries, although such benefits in Japan were notably modest. Elevating Japan’s cash benefit scheme could potentially lead to higher TFR.

**Funding:**

HIAS Health, 10.13039/100030883Hitotsubashi University; Tokyo Foundation for Policy Research, Japan.

**Research in context**

**Evidence before this study**

Studies have demonstrated that cash benefit policies paired with childcare coverage can increase the fertility rate in several high-income countries. While Japan and Korea have experienced substantial fertility declines over past decade, public spending on family benefits was still comparatively low in comparison to most of the OECD countries. To assess whether fertility rates in Japan could be reversed by 2030, our research began with a comprehensive review of public policies that could potentially have an effect. We then applied this policy to the Japanese situation. To identify relevant studies evaluating the impact of population-level policies on fertility rates, searches were conducted in the PubMed, Web of Science and CINHAL databases from inception to September 11, 2023 without language restrictions. We initially searched PubMed using the following terms: (cash benefits OR cash transfers OR allowances OR baby bonuses OR parental leave OR service coverage OR childcare coverage OR financial support OR tax exemption OR parental leave OR maaternity leave OR public policies OR policies) AND (total fertility rate OR TFR OR fertility rate OR birth rate OR parity OR birth) NOT (“low-income countries” or “lower-middle-income countries” or “developing countries”) (details in Appendix p2). 2484 relevant articles were identified, of which 131 were reviewed in full-text and 53 were eligible for our study. Our comprehensive database search identified several systematic review studies relevant to fertility rates and public spending on family benefits. Although several systematic reviews attempt to provide information on effective fertility policies, they all have some limitations, such as focusing on specific regions or specific policies, such as cash incentives or maternity leave. Furthermore, none of the studies examined how effective policies impact future fertility rates in countries with continuously declining fertility and increasing older population, especially Japan. The information will help national policy planners design and reform Japanese fertility policies.

**Added value of this study**

This is the first comprehensive systematic review and scenario-based secondary data analysis using Bayesian regression models. The systematic review included 47 studies and identified that the cash benefit (payment at birth, allowances, maternity leave), service benefit (childcare coverage) and tax refund policies were the most influential policies for increasing fertility rates in high income countries. Our study found that France had the highest fertility rate in 2022, with 1.80 births per woman. Sweden, Australia, the UK, and Hungary also had higher rates than the OECD average of 1.58. Korea and Japan had the lowest, at 0.84 and 1.26, respectively. Governments in Japan, Germany, Greece, France, and Hungary increased service benefits, while cashtransfers in Australia, Greece, Hungary, France, and the UK exceeded 1.3% of GDP. Length of maternity leave ranged from 14 weeks up to 43 weeks in Greece and 24 weeks to 16 weeks in Hungary. Correlation analysis showed a positive relationship between cash and service benefits and fertility rate, but no link between duration of maternity leave and TFR. According to Bayesian analysis, if Japan maintains its current cash benefit policy (0.74% of GDP), the chances of reversing the declining fertility rate by 2030 is 12%, and by 2035 is 29%. Imitating France, Hungary and Australia’s cash benefit policies (1.50%, 1.72% and 1.66% of GDP, respectively) by 2030, would increase the probability of reversing the fertility rate in to higher than 69%, 70%, and 69%, respectively. Reversing the fertility rate by 2035 would also increase the chances to around 65%, 68%, and 79%, respectively. These results suggest that policy implementation sooner rather than later will likely lead to a faster and more likely rise in the fertility rate in Japan.

**Implications of all the available evidence**

The available evidence, coupled with Bayesian scenario-based model derived from this study, underscores how family benefits especially cash transfer policy affect on future fertility in Japan. In order to reverse Japan’s declining fertility trends, strategic adjustments in cash benefits are crucial. A successful policy can be transferred across borders to address decline fertility rates, which has implications not only for Japan but also for the rest of the world.

## Introduction

Nearly half of the world’s population (83 out of 201 countries) now lives in countries where total fertility rate (TFR) is below the replacement threshold of 2.1 births per woman.^1^ Low fertility is no longer confined to high-income countries but has become a global issue, though rates vary significantly. For example, the average TFR in OECD countries is approximately 1.58 births per woman in 2021, with Korea and Japan recording notably lower rates of 0.81 and 1.30, respectively.^2^^,^^3^ A recent study predicted a potential decline in Europe’s population, with Japan expected to experience a nearly two-thirds reduction by 2100.^4^ The convergence of declining fertility rates, decreasing mortality, and increasing life expectancy are the major drivers to this downward trend. To counteract these demographic shifts, many countries have adopted policies aimed at increasing fertility rates.^5^, ^6^, ^7^, ^8^, ^9^, ^10^, ^11^, ^12^, ^13^, ^14^ As of 2019, nearly three-quarters of the world’s governments had fertility-related policies,^15^ with 69 aiming to reduce fertility, 55 to increase fertility, and 19 to maintain current levels. Given the pressing nature of the demographic changes, particularly the issues arising from low fertility rates, there is a critical need for updated, evidence-based research to evaluate the impact of policies designated to boost fertility and to scale up successful interventions to effectively address the current demographic crisis effectively.

High-income countries have implemented various family benefits policies to tackle the fertility decline and alleviate the financial burdens on families. These policies include: cash benefits (such as allowances, cash transfers, paid leave, birth payments), service benefits, and financial incentives like refunds or exemptions.^5^, ^6^, ^7^, ^8^, ^9^, ^10^, ^11^ Generally, more generous and expensive family benefit policies are considered to boost fertility rates.^5^, ^6^, ^7^, ^8^ However, the effectiveness of these policies varies, showing positive, negative or no impact on fertility rates. While some studies indicate that cash benefits and parental leave positively influence fertility,^9^, ^10^, ^11^ others have not observed this effect.^12^ Another study shows that in Norway, Sweden and Finland, a mixed relationship between paternal leave and fertility rate, with fathers taking parental leave is positively association with an increase in second births, though this trend does not extend to third births in Norway and Sweden, illustrating the complex relationship between family benefit policies and fertility outcomes.^16^ A recent systematic review by Bergsvik et al. found that childcare expansions positively affect fertility rates, while the impact of increased cash transfers is typically temporary.^17^ Various police, such as universal child allowances,^9^ baby bonuses, and parental leave,^10^^,^^16^ and multi-component benefit programs have been connected associated with higher fertility rates in high-income countries.^9^^,^^18^ In contrast, tax exemptions, have demonstrated a limited impact.^13^^,^^14^ It is critical to recognize that these systematic reviews were conducted before 2020 and focused on assessing specific policies. This context underscores the need for a comprehensive and updated synthesis of research on fertility policies. Such a synthesis would be instrumental in identifying the most effective strategies to counter declining fertility rates in advanced economies, ensuring that policy interventions are grounded in the latest and most comprehensive available evidence.

In the Pacific region, particularly in Japan and Korea and even in China, there is a strong focus on initiatives aimed at boosting fertility rates, responding to a pressing demographic challenge characterized by persistent decline in fertility rates, with significant impacts on societal structure and economic stability. Over the past 30 years, Japan and Korea have experienced substantial reductions on in their total fertility rates (TFRs), with Japan’s TFR decreasing from 1.54 in 1990 to 1.26 in 2022, and Korea’s TFR declining from 1.57 to 0.78 over the same period. The ongoing decline contributes to an aging population, a shrinking workforce and reproductive-age population, and potential economic challenges ahead.

Despite these trends, public funds allocated to family benefits, especially cash transfers, remain relatively low in Japan and Korea compared to other OECD countries like Australia, France, Hungary, Germany, and the United Kingdom.^3^ The government’s efforts in Japan to elevate fertility through child allowances, childcare service, and parental leave have yet to successfully counteract the fertility decline (Box 1). Therefore, a comprehensive and extensive assessment of both domestic and international fertility policies, coupled with a detailed evaluation of their potential outcomes, is essential to determine whether Japan’s declining fertility rate can be reversed. The objectives of this study were twofold: first, to identify effective policies that have successfully reversed fertility declines through a systematic review, and second, to apply these effective policies to Japan in order to forecast TFR up to the year 2035.

Box 1 **Fertility policies in Japan.**

Japan lacks a comprehensive policy package to address the issue of the declining birthrate. Instead, the government has implemented individual measures such as child allowances, parental leave, and the establishment of childcare facilities.

Child Allowance program is a financial assistance initiative aimed at supporting families having children. It was established in 1946 as a government effort to rebuild the country after World War II. Initially targeting parents with two or more children under 15, it later expanded to cover all families with children under 18. This program aims to provide financial assistance to families, helping alleviate the financial burden of raising children and promoting the overall well-being of families. Children who are eligible receive a Childcare Allowance of 10,000 yen to 15,000 yen (approximately $88 to $177) per month.

Parental leave benefits in Japan provide financial support and guidance to employees during the crucial period before and after childbirth. Women receive a special salary payment during their maternity leave, which is two-thirds of their average earnings, and men are eligible for a similar payment during their paternity leave. Additionally, both mothers and fathers are entitled to a one-time cash payment of approximately JPY 300,000 to help cover medical expenses and initial childcare costs. The implementation of these benefits has evolved over time, with key milestones including the Maternity Protection Act in 1954, the Parental Leave Act in 1961, and further revisions in 1987 and 1991. The latest update in 2019 further promotes parental leave benefits and gender equality in the workplace, demonstrating Japan’s commitment to supporting working parents and their family responsibilities.

Childcare facilities have a rich history dating back to the immediate aftermath of World War II. Since then, policies have shifted towards both the quantitative and qualitative expansion of these facilities, with the goal of making childcare more affordable and eliminating waiting lists. Some municipalities have also implemented fee exemptions for households with multiple children, and free kindergarten and nursery admissions have been introduced with certain subsidies provided by local authorities. However, challenges such as low wages for childcare workers, the need for uniform improvement in the quality of childcare, and the burden of childcare fees for multiple children still exist.

As a comprehensive plan for supporting children and families, Kishida announced the Child Future Strategy in December 2023. As part of its "children’s future strategy,” the government has allocated 3.6 trillion yen (approximately $25 billion). This comprehensive package will provide enhanced benefits to both working and nonworking parents and support families in their role as caregivers. As a result of these plans, child allowances will be increased in the next year as well. By 2030, the government intends to implement measures to reverse the declining birth rate trend. Natural childbirth expenses will be covered by the policy starting in October 2024. The reform will include the removal of the current allowance income cap and will entitle all parents with children up to high school age to a monthly payment of ¥15,000 for the first two children aged up to 2 years and ¥10,000 for children aged 3 to high school age. For third and subsequent children, the amount will increase to ¥30,000 per child throughout high school. Moreover, the Child Future Strategy provides tuition-free university education to ensure equality for all students. Specifically, dual-income households will benefit from 100% parental leave subsidies and 10% childcare subsidies. In fiscal 2025, paternity leave benefits will be increased, encouraging shared parenting. These reforms will encourage childbirth and stabilize the population by reducing financial burden.

## Methods

### Data and methods

Our study employed two data extraction approaches. In the first step, we identified relevant studies that assessed the impact of various policies on fertility rates.

### Systematic review

In the first step, a systematic review was done by searching several databases including PubMed, Web of Science, and CINHAL from inception to September 11, 2023, without imposing language restrictions. The search strategies were further updated and extended to include studies published between September 12, 2023 and March 27, 2025. The search strategy encompassed terms: (cash benefits OR cash transfers OR allowances OR baby bonuses OR parental leave OR childcare coverage OR financial support OR tax exemption OR parental leave OR maternity leave OR policies) AND (total fertility rate OR fertility rate OR birth rate OR parity). Our search strategy, study selection, and evidence synthesis are detailed in the Supplemental appendix (eMethod S1 and Supplemental Tables S1–S3, pp.2–5). The systematic review protocol is registered with PROSPERO (ID: CRD420251020541).

Two researchers were independently review the titles and abstracts of the studies retrieved during the search to assess whether they meet the inclusion and exclusion criteria. If there are disagreements, a third researcher will be consulted. The full text versions of the selected articles will also be read by two researchers, with disagreements once again resolved by a third researcher if necessary. Finally, two authors will independently review the articles included, assessing the intervention’s nature, methodology, sample size, country, policy, outcomes, time period, outcome measure, type of data, and control variables. Any discrepancies will be discussed or a third researcher consulted. The selection of study was performed based on PRISMA flowchart (appendix Supplemental Fig. S1). Two independent authors extracted range of data from the included studies such as author name, publication year, survey year, country, settings, sample size, name of policy, policy description, policy implementation time, name of outcome, definition of outcome, key results, and impact and results directions per policy.

### Study quality assessment

The quality (or risk of bias) of observational studies was assessed using the Newcastle–Ottawa Scale,^19^ while controlled before-and-after studies, interrupted time series, and other quasi-experimental designs were evaluated using the Cochrane EPOC criteria.^20^ Studies were categorized based on total scores as high (≥6), moderate (4–5), or low quality (0–3). Further details on the quality assessment process are provided in the supplementary material (appendix eMethod S2, pp. 3–4). Two reviewers independently conducted the assessments, with results cross-checked by two additional authors. Any discrepancies were resolved through discussion.

### Secondary data analysis

In the second step, we did a series of scenario-based projections for Japan’s TFR from 2024 to 2035. These scenarios were based on the most effective fertility policies that were identified from the systematic review. For the data analysis in this second step, we extracted a comprehensive set of data from the OECD database, which included metrics such as TFR, mean age at first birth, marriage rate, women’s employment rate, family benefit spending, proportion of young population, and maternity leave details. The proportion of the population aged 0–14, expressed as a percentage of the total population, was obtained from the United Nations (UN) World Population Prospects (WPP). Additionally, we gathered GDP per capita data from the International Monetary Fund (IMF) database. Further details on the OECD database can be found at this link: http://www.oecd.org/els/family/database.htm, while an IMF profile on GDP per capita can be found here: http://www.imf.org/external/datamapper/profile/JPN.

### Variables

The primary outcome variable in this study was fertility rate, including TFR, birth rate, birth order, and parity progression. To forecast TFR up to 2030 or 2035, we considered several predictor variables, including mean age at first birth, maternal employment rate, marriage rate, GDP per capita, proportion of population aged 0–14 years, and the proportion of GDP allocated to service and cash benefits. Cash benefits are defined as financial transfers to families with children, payments at birth, allowances, parental leave payments, and income support for single-parent families. Public spending on services for families with children includes subsidies for childcare, early childhood education, and youth assistance. Support on family services, such as centre-based facilities and home help, is also included. A detailed list and overview of public spending on family benefits are available in the Supplemental appendix (Supplemental Table S4, p.8). Descriptions of the predictor variables are presented in the Appendix (eMethod S2, p.9).

### Statistical analysis

Bayesian hierarchical regression models were applied to estimate trends and project Japan’s Total Fertility Rate (TFR) from 1990 to 2035. The model employed a logit transformation of TFR as the dependent variable, with predictors such as cash benefits, GDP per capita, proportion of young population (014 years), marriage rate, mean age at first marriage, and unemployment rate log-transformed and mean-centered for interpretability. The marriage rate was included as a predictor due to its strong correlation with childbirth trends in Japan, where out-of-wedlock births remain rare. The unemployment rate was incorporated to capture the impact of economic stability on fertility decisions, as financial insecurity can act as a deterrent to childbearing. Counterfactual scenarios simulated policy changes by adjusting cash benefits to levels observed in countries like France and Germany, while other factors remained constant. Posterior predictive distributions accounted for uncertainty, enabling scenario-based projections to assess policy impacts. Cultural and structural factors were treated as time-invariant, implicitly shaping policy effectiveness. This approach isolates the demographic effects of cash benefits under various policy regimes. The Bayesian hierarchical regression model is specified as follows:

*Likelihood:*

$$y_{i}=N\left(\mu_{i},\sigma^{2}\right)$$

$$\mu_{i}=\beta_{0}+\beta_{1}*X_{1i}+\beta_{2}*X_{2i}+\beta_{3}*X_{3i}+\beta_{4}*X_{4i}+\beta_{5}*X_{5i}+\beta_{6}*X_{6i}+\beta_{7}*X_{7i}$$

Where:

$X_{1i}$: Year for observation i

$X_{2i}$: Cash benefits for observation i

$X_{3i}$: Proportion of populaiton aged 0–14 years

$X_{4i}$: GDP per capita for observation i

$X_{5i}$: Unemployment rate for observation i

$X_{6i}$: Marriage rate for observation i

$X_{7i}$: Mean age at first birth for observation i.

$\beta_{0}$ Intercept; $\beta_{1},\ldots ,\beta_{7}$: Regression coefficients for predictors; and $\sigma^{2}$: Variance of the error term

*Prediction:*

$$a.tfr\_pred\left[i\right]=100*\frac{\exp\left(\mu \left[i\right]\right)}{1+\exp\left(\mu \left[i\right]\right)}$$

*Threshold:*

The model evaluates whether predicted TFR exceeds a threshold (e.g., 1.3):

$$b.tfr.crit\left[i\right]=1\left(a.tfr_{pred\left[i\right]}>1.3\right)\text{.}$$

Where: 1 is the indicator function.

*Assumptions:*

1. Linear relationship: The logit-transformed TFR depends linearly on the predictors (year, cash benefits, and other covariates)
2. Normmality: The errors ($y_{c}\left[i\right]-\mu \left[i\right]$) are normally distributed with mean 0 and precision $\tau =\sigma^{-2}$
3. Centering: All covariates are mean-centered for better convergence and interpretation of $\beta_{0}$ (the intercept)
4. Priors: The regression coefficients ($\beta_{0},\beta_{1},\ldots ,\beta_{7}$) have weekly informative Gaussian priors: $\beta_{j}∼N\left(0,100\right)$ ; and the error variance ($\sigma^{2}$): σ ∼Half-Cauchy (0.0.04)
5. TFR is transformed using a logit function for bounded outcomes (0–100)

The model incorporates counterfactual scenarios by adjusting $X_{2i}$ (cash benefits) to match levels observed in other countries (e.g., France, Germany, Hungary) while keeping other predictors constant. This approach evaluates the impact of enhanced financial incentives on Japan’s fertility rates under various policy regimes.

The first scenario, termed the “Japan current policy” scenario, utilized covariates including year, cash benefit as a percentage of GDP specific to Japan, proportion of populaiton, mean age at first birth, marriage rate, female employment rate, and GDP per capita. For this scenario, the model presumed that Japan’s cash benefits from 2023 to 2035 would remain constant at the 2022 level. All predictors in the current scenario model were tailored to the Japanese context.

Subsequently, we constructed additional scenario-based projections for Japan’s TFR up to 2035, integrating various international cash benefit policies. We selected reference countries and their associated impactful fertility policies based on our systematic review and correlation analysis. Consequently, Australia, France, Germany, Greece, and Hungary were chosen as benchmark countries to inform the projections for Japan’s future TFR, illustrating how adopting similar policies might affect Japan’s fertility rates.

In the second scenario, we also incorporated the most recent cash benefit data from the selected countries. This recent cash benefit was determined by calculating the average cash benefits over the last 10 years for each country. The aim was to set these averages as Japan’s projections from 2024 to 2035, the cash benefit values were calculated using a linear interpolation approach, taking into account the international policies (appendix Supplemental Table S5, p.10).

The Bayesian model for this analysis was fitted with five chains for 10,000 iterations each, incorporating a burn-in period of 1000 iterations and a thinning rate of 10 to ensure sample independence and adequate mixing. We used trace plots and the Gelman-Rubin diagnostic statistics to monitor the convergence status in order to ensure the chains had converged to a stable solution. We conducted a sensitivity analysis by altering priors and excluding the key predictor, marriage rate, to compare results. The model’s predictive performance was evaluated using Leave-One-Out Cross-Validation (LOO-CV), with the LOO Log Predictive Density (LOO-LPD) as the key metric.^21^ Higher LOO-LPD values indicate better predictive accuracy. This approach allowed for a direct comparison of model performance between the 2030 and 2035 scenarios to assess improvements in assumptions and projections. To further validate the model, a posterior predictive check was conducted to ensure the predictions closely matched the observed data, confirming the model’s reliability and fit.^21^

### Role of the funding source

The funding for the research was provided by HIAS Health, Hitotsubashi University and Tokyo Foundation for Policy Research, Japan. The funder had no role in study design, data collection and analysis, decision to publish, or preparation of the manuscript.

## Results

### Evidence of effectiveness

Our electronic database search identified 3005 relevant articles pertinent to our research topic for initial screening based on titles and abstracts. Subsequently, 158 studies were subjected to full-text review and 61 studies met the eligibility criteria for our study (appendix Supplemental Fig. S1, p.11). These studies, published between 1992 and 2024, encompassed research from 16 individual countries, and 8 multi-countries, predominantly focusing on OECD countries. Our systematic review found that cash benefits were the most frequently reported fertility policies in 57 studies. This was followed by service benefits in 9 studies and financial incentives in 7 studies. The cash benefit policies most frequently examined were child allowances, paid parental leave, birth payments, and paid maternal leave (Supplemental Table S6, pp.12–19). The majority of studies were assessed as high quality (n = 49), while only a small number were rated as moderate (n = 7) or low quality (n = 5) (Appendix Supplemental Table S6, pp.12–19).

Table 1 presents a detailed narrative summary of the results from all the included studies, according to fertility policies and their observed impacts—whether significantly positive, significantly negative, or insignificant with either an increase or decrease. The effectiveness percentage for each policy was calculated by the ratio of studies with positive and significant results to the total number of studies on each specific policy and outcome. Policies were classified as “highly effective” if their effectiveness percentage was more 80% or higher, “effective” for effectiveness percentages between 60 and 80%, “moderately effective” for those between 40 and 60%, “low” for percentages between 20 and 40%, and “very low” for those between 0 and 20%. In our study, policies such as payments at birth, allowances, paid maternity leave, childcare coverage, and tax exemptions were among the most effective policies to increase fertility rates. Paid parental leave, Assisted Reproductive Technology health insurance coverage, and China’s universal two-child policy was also identified as effective. While tax refunds and unpaid maternity leave were considered effective, additional evidence is needed to draw a conclusion.

**Table 1:** Narrative summary of fertility change following the implementation of different fertility policies (N = 61 Studies).

Fertility polciy	# Studies	Effective policy (%)^d^	Outcomes					
			Fertility rate^e^			Pregnancy rate		
			Sig (+)	Sig (−)	No	Sig (+)	Sig (−)	No
**Cash benefits**	57							
Cash benefits (at birth)	19	89%	17	1	1			
Cash benefits (allowance)	15	93%	14		1			
Cash benefits (paid parental leave)	21	62%	13	1	7	2		
Cash benefits (unpaid maternity leave)	2	100%	2			2		
**Service benefits**	9							
Childcare coverage	9	89%	8		1			
**Financial support**	9							
Tax examption	5	100%	5					
Tax refund	4	75%	3	1				
**Mixed benefits**	3							
Mixed benefits^a^	1	100%	1					
Mixed benefits^b^	1	100%	1					
Mixed benefits^c^	1	100%	1					
ART health insurance coverage	2	100%	2					
**China’s two child policy**	3	100%	3					

Note: Each cell shows the number of studies. Sig (+) indicates a statistically significant positive effect on fertility outcomes, Sig (−) indicates a significant negative effect, and No indicates no statistically significant effect. The total number of studies may exceed the sum across policy categories, as some studies evaluated multiple policies with the same outcome.

ART, Assisted Reproductive Technology.

a Family allowance, tax relief, childcare fee, payment at birth, monthly maternity allowance, monthly allowance for nonworking mothers.

b Paid parental leave benefit and child care coverage.

c Payment at birth and paid parental leave.

d To calculate the effective policy percentage, we divided the number of studies with positive significant results by the total number of studies on each outcome and policy. For example, the percentage of effective cash benefits (at birth) is 86% (12/14). We then categorize the policy from highly effective to low effective depending on this effective percentage. For example, the fertility policy can be divided into five equal parts based on their level of effective percentage: 0–20% (very low effective), 20–40% (low effective), 40–60% (moderate effective), 60–80% (effective), and 80–100% (highly effective). When there are only three or fewer studies, conclusions need to be cautiously drawn. Here, we level the effeitive class, but need more evidence.

e Fertility rate includes total fertility rate, general fertility rate, complete fertility rate, birth rate, number of children/births.

Table 2 presents the summary results of the impacts of various fertility policies across major demographic segments, with Supplemental Table S7 providing the detailed results (Appendix, pp. 20–21). In 2004, Australia’s introduction of a one-time birth incentive led to a 12.8% increase in fertility rates. Similarly, Spanish government’s one-time birth incentive of Euro 2500 per birth from 2007 to 2010, increased fertility by 4.7% until 2011. However, when the policy was canceled, fertility decreased by 5.7%. Canada’s quarterly birth incentive payments, implemented between 1988 and 1997, and ranged from $500 for a first child to $8000 for a third, were associated with successful fertility increases by 4.4 and 9%.^27^ Women between age 25 and 40 years witnessed the most significant impact^27^, ^28^, ^29^, ^30^, ^31^^,^^33^^,^^37^ due to these policies. However, a study in Australia^23^ revealed that the both payments had a significant impact on mothers aged 20–24. Additionally, studies conducted in different countries, such as Germany, Australia and the USA, highlighted the positive impact of parental leave benefits, birth payments, and cash allowances on fertility rates, particularly among women aged 35 and over.^22^^,^^28^, ^29^, ^30^, ^31^^,^^34^^,^^35^ Furthermore, the influence of educational attainment on fertility responses to various family policies shows a significant variation. For instance, parental leave benefits and childcare support tend to increase fertility rates more substantially among women with higher education than among those with middle or lower educational levels.^9^^,^^16^^,^^22^^,^^38^ In contrast, the fertility impact of birth payments dropped by 19.2% among women with a university degree compared to lower educated women.^27^ In Australia, the introduction of child subsidies resulted in a 2.2% increase in third-child births.^23^ In Hungary, cash benefits for second, third, and fourth births led to increases in fertility by 26%, 32%, and 14%, respectively.^32^

**Table 2:** Impact of policy on fertility rates by age, education and parity.

Policy	Country (Study)	Age	Education	Parity/birth order
Parental leave benefit	Germany (Raute, 2019)^22^	Parental leave reform induced increases in fertility (>5%) for women aged 35–39 and 40–44 years old.	Parental leave reform increased fertility by close to 23% for highly educated, working women, and by 9% for medium-educated women.	NA
	Sweden and Norway (Duvander, 2020)^12^	NA	Parental leave benefits in Sweden and Norway were associated with higher parity (2nd and 3rd), particularly among highly educated women.	Parental leave benefits in Sweden and Norway were associated with higher parity (2nd and 3rd) births, particularly among highly educated women.
	Iceland (Einarsdottir, 2023)^23^	A decrease in parental leave also led to a decrease in birth rates in the 25–34 age group (15.8%), and an increase in parental leave led to an increase in estimated births (9.3%). Women aged 35–44, on the other hand, did not see much change in birth rates.	NA	NA
Cash benefit (payment at birth)/baby bonus	Australia (Einarsdóttir et al., 2012).^24^	Cash benefits at birth increased fertility the most in mothers aged 20–24 years (26.3%), followed by mothers aged 25–29 (14%).	NA	Cash benefits at birth increased third and fourth parity by 1.6% and 2.2%, respectively.
	Australia (Chen et al., 2018).^25^	Cash benefits at birth had a greater impact on married women aged 35–39	NA	Cash benefits at birth had a greater impact on parity 2 or 3 parity women compared to zero parity women.
	Australia (Langridge et al., 2012).^26^	Women aged 20–24 saw a fertility increase of 28.1 births per 1000 in 2005–2006, compared to 13.5 births per 1000 for those aged 25–29	NA	Fertility rates were highest in major cities, growing from 32.4 in 2004 to 38.8 in 2006. Outer regional areas had the biggest rise, increasing by 5.9 points from 2004 to 2005, followed by inner regional areas with a 4.3-point rise from 2005 to 2006.
	Canada (Milligan, 2005).^27^	Kim (2014) found birth subsidies had a decreasing effect on fertility with age, with only a 0.8% increase in fertility for the oldest cohort. At 6.6% and 4.2%, the impact is highest for the youngest and second-youngest cohorts.	Women with a university degree were estimated to have a 19.2 percentage point lower likelihood of having a child.	Cash benefits at birth increased the possibility by 10% for first births, by 13% for second births, and by 25% for third births, given that the last group received the highest subsidies.
	Poland (Bokun, 2024)^28^	Cash transfers were linked to increased fertility among women aged 31–40 (by 0.7–1.8 percentage points), but decreased fertility among those aged 21–30 (by 2.2–2.6 percentage points).	Cash transfer showed no significant effect on fertility for women with primary (β = 0.002, 95% CI: −0.017 to 0.021, p = 0.84) or postsecondary education (β = −0.002, 95% CI: −0.009 to 0.006, p = 0.70).	NA
	South Korea (Hong et al., 2016).^29^	Cash grants at birth increased crude birth rates most for mothers aged 30–34 (3.79 units), followed by ages 25–29 (2.49 units) and 20–24 (1.29 units).	Cash grants at birth increased the crude birth rate more in urban districts (5.52 units) than in rural areas (1.78 units).	Cash grants at birth had more impact on 1 parity (1.9 units) and 2 parity (0.65 units) women compared to others.
	South Korea (Son, 2018)^30^	Family benefits have a positive effect on most age-specific fertility rates, with the greatest impact observed among women aged 35–39, showing a 5.9% increase, followed by women aged 40–44, with a 5.5% increase.	NA	NA
	South Korea (Kim, 2024)^31^	The effect of cash transfers on birth rates is strongest for women aged 25 to 30, with increase of up to 0.2 for first births, but declines steadily thereafter, approaching zero by age 40	NA	Cash transfers have the strongest positive impact on first births (up to 0.2), a moderate effect on second births (around 0.1), and a negligible impact on third births (close to 0).
	USA (Cowan et al., 2022)	Women aged 15–24 years received cash benefits (annual allowances) but the effects were not statistically significant. For women aged 25–29 and 30–34 years, we saw bigger effects than for women 35–39 and 40–44 years.	NA	Cash benefits (annual allowances) had a very large effect on birth rates for the first and second births, but no effect on the third births, and a very small effect on the fourth births.
Cash benefits (at birth, allowance, paid maternity leave)	Hungary (Gabos et al., 2009)^32^	NA	NA	A significant association existed between receiving cash benefits (at birth, allowances, and paid maternity leave) and having a second, third, and fourth child (26%, 32%, and 14%, respectively).
Childcare coverage	Germany (Bauernschuster et al., 2015).	NA	Association between childcare coverage and female education was positively and strongly associated (p < 0.05) which shows that the policy had positive effects on fertility, driven mainly by women in the upper-end of education.	NA
	Japan (Fukai, 2017)^33^	Increases in childcare availability from 2000 to 2010 led to a small but significant increase in the fertility rate of women aged 25–39 living in regions where women tend to work more, but had no significant effect in other regions.	NA	NA
	16 European countries (Baizan et al. 2016)^9^	NA	Childcare coverage and fertility are positively related for all educational groups, but this relationship is stronger for the highly educated (p < 0.01) and weaker for the lower-educated (p < 0.1), as compared with women with a medium level of education.	NA
Tax exemption	USA (Ridao-cano et al., 2005)^34^	Women aged 20–24 years (p < 0.05) were more responsive to the tax exemption policy than women aged 25–34 years (p < 0.05) in terms of fertility.	NA	NA
Two-child policy	China (Chen et al., 2022)^35^	The monthly percentage of births to mothers aged 35 and older rose significantly from 7.39% during the one-child policy period to 10.59% during the partial two-child policy period, and further to 13.65% during the universal two-child policy period.	NA	During both the partial and universal two-child policy periods, the monthly percentage of births to multiparous women consistently exceeded that of nulliparous women.
	China (Li et al., 2019)^36^	The monthly percentage of births to mothers aged ≥ 35 increased significantly by an additional 5.8 percentage points during the universal two-child policy period.	NA	During the universal two-child policy, births to multiparous women exceeded nulliparous women by 9.1 percentage points.

### TFR trends in OECD countries

**Fig. 1:**
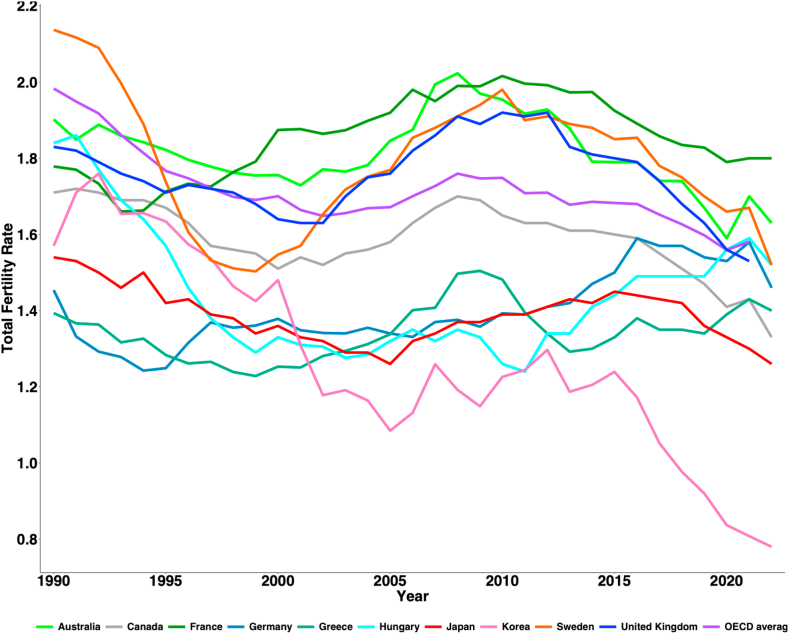
Total fertility rate in selected OECD countries, 1990–2022.

Fig. 1 shows the trends in TFR in selected OECD countries from 1995 to 2022. In 1990, Australia’s TFR was 1.90, declining to 1.76 in 2000, then increasing to 2.02 in 2008. However, it dropped again to 1.59 in 2020 and slightly recovered to 1.63 by 2022. In contrast, France’s TFR increased from 1.78 in 1990 to 1.87 in 2000, peaking at 1.99 in 2008, then dropping to 1.79 in 2020 and slightly increasing to 1.80 in 2022. Throughout the observed period, both Canada and Germany maintained TFRs below the OECD. Hungary’s TFR dropped from 1.84 in 1990 to 1.33 in 2000, remained relatively stable until 2008 (around 1.30), and then showed a growth trend, reaching 1.59 by 2021. Japan and Korea both experienced consistently declining TFRs from 1990 to 2005, with subsequent increases between 2005 and 2015. Specifically, Japan’s TFR decreased from 1.54 in 1990 to 1.26 in 2005. From 2006 to 2015, there was a modest rise to 1.45 in 2015, followed by a decrease to 1.30 in 2020. In 2022, South Korea recorded the lowest TFR of all OECD countries at 0.78, followed by Japan (1.26), Canada (1.33), Greece (1.40), and Germany (1.46). Conversely, France had the highest TFR at 1.80, followed by Sweden (1.52), Australia (1.63), the United Kingdom (1.67), and Hungary (1.52), all of which were above the OECD average of 1.58.

Data source: OECD database [https://www.oecd.org/els/family/database.htm].

### Association between family benefits and TFR

**Fig. 2:**
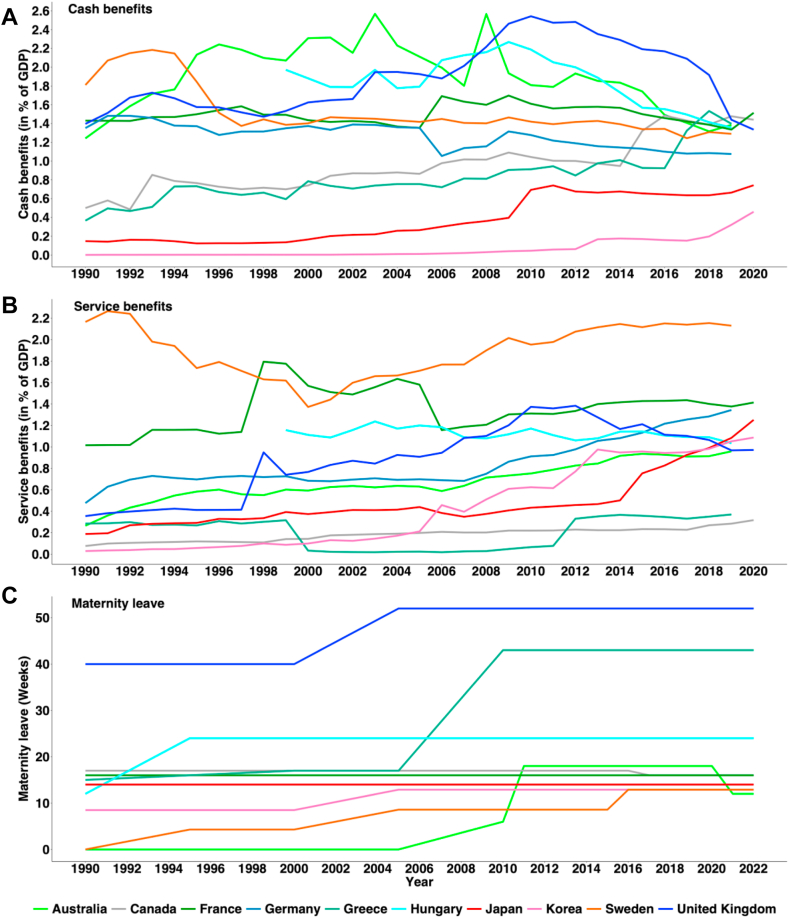
Public cash benefits (A) and service benefits (B) and maternity leave duration (C) in some selected OECD countries, 1990–2022. Note: Family benefits spending as a percentage of GDP from 1990 to 2020 has been tracked. This includes cash transfers for children, income support for single parents, and subsidies for childcare. Services include childcare support, young people assistance, home help, and centre-based facilities.

**Fig. 3:**
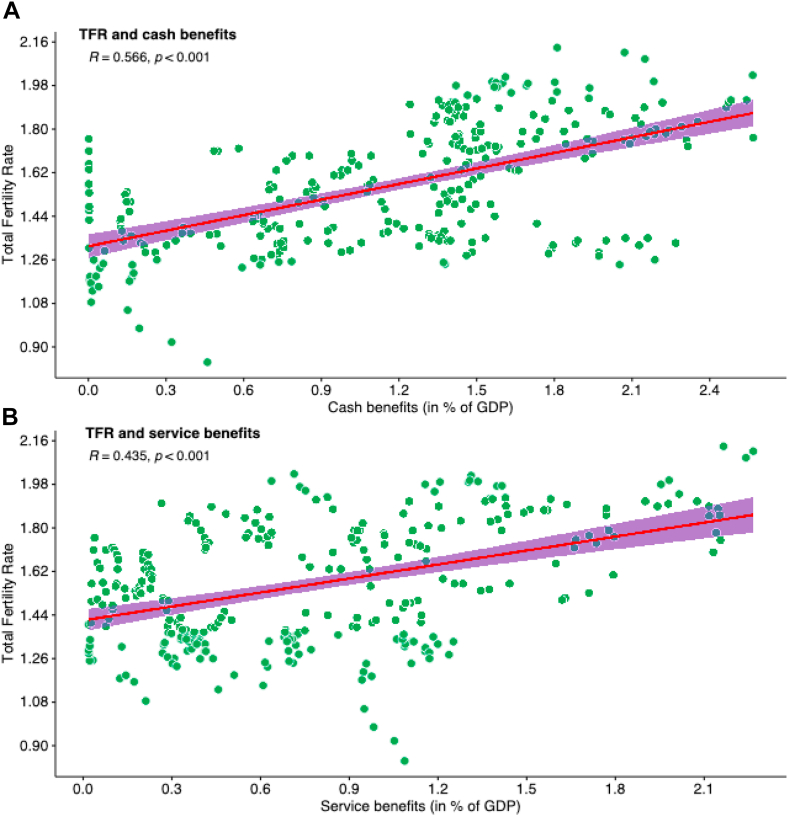
Correlation between total fertility rate and family service benefits, 1990 to 2020. TFR, total fertility rate; R, correlation coefficients; Data sources: OECD database. Note: We explored the relationship between TFR and family benefits using OECD data from 1990 to 2019. Only Australia, Canada, France, Greece, Germany, Hungary, Japan, Sweden the United Kingdom were included in this correlation analysis. (A) shows the correlation between total fertility rate (TFR) and cash benefits, and (B) shows the correlation between TFR and service benefits.

Fig. 2 demonstrate the trends in cash benefits (2. A), childcare services (2. B), and duration of maternity leave (2. C) from 1990 to 2019. In 2019, the average cash payments were 1.37% of GDP across Germany, Greece, France, and Hungary. Notably, countries like Australia, Greece, Hungary, France, and the United Kingdom allocated more than 1.3% of their GDP to family cash transfers. Japan experienced a small increase in cash benefits over three decades, starting from 0.15% of GDP in 1990, increasing to 0.17% in 2000, and reaching 0.69% in 2010, then slightly decreasing to 0.66% in 2019. Throughout the year, the service benefits expanded in many countries, including Sweden, France, Germany, Hungary, Korea, and Japan from 2000 to 2020 (Fig. 2B). Conversely, Canada and Greece had the lowest levels of service benefits. Specifically, Japan’s service benefits increased from 0.19% of GDP in 1990 to 1.08% in 2019. In the same time period, Germany’s service benefits increased from 0.48% to 1.34%, Greece’s from 0.28% to 0.37%, France’s from 1.02% to 1.38%, and Hungary’s decreased from 1.16% to 1.03%. In terms of maternity leave benefits, most countries including Japan offered around 14 weeks in 2019 (Fig. 2C). However, Greece provided 43 weeks, Hungary 24 weeks, and France 16 weeks of maternity leave. Additionally, we analyzed the relationship between TFR and family benefits using OECD data from 1990 to 2019 (Fig. 3). This correlation analysis included only Australia, Canada, France, Greece, Germany, Hungary, Japan, Sweden and the United Kingdom. The analysis showed that both cash and service benefits have a significantly positive correlation with TFR (cash benefits: correlation coefficient = 0.566 and p < 0.001 in Fig. 3A; service benefits: correlation coefficient = 0.435 and p < 0.001 in Fig. 3B). However, we found no significant correlation between duration of maternity leave and TFR (correlation coefficient = 0.035 and p = 0.95 in appendix Supplemental Fig. S2, p.19). The detailed country specific correlation plots are presented in the appendix (Supplemental Figs. S3 and S4, pp. 22–23).

Data source: OECD database [https://www.oecd.org/els/family/database.htm].

### Scenario-based TFR projection in Japan

**Fig. 4:**
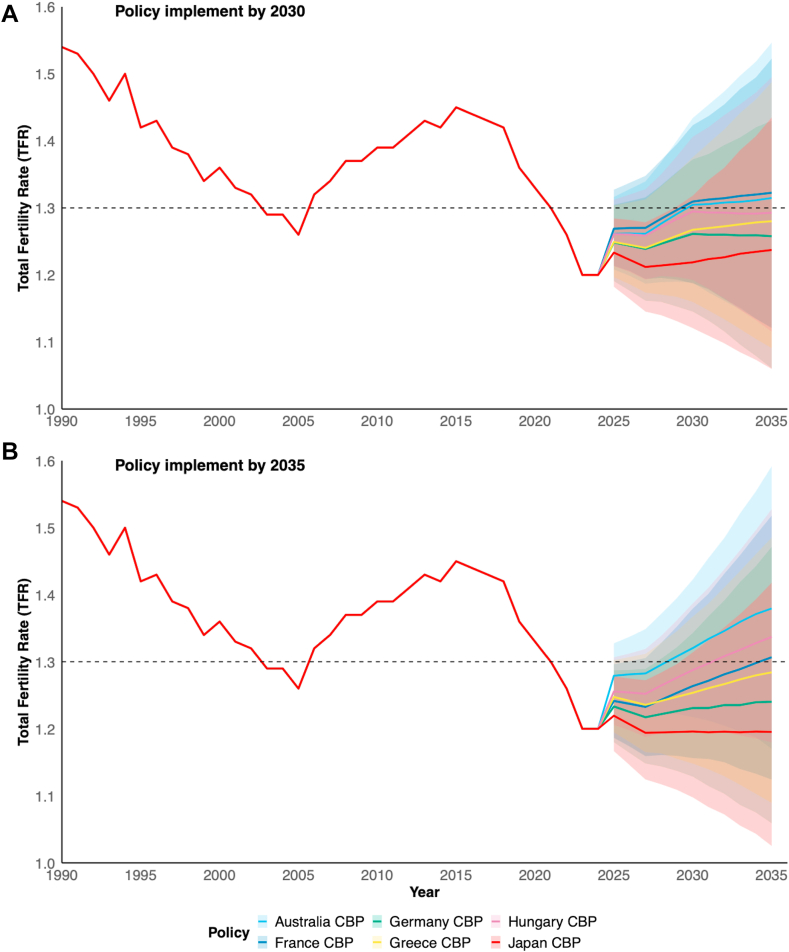
Scenario-based fertility projection in Japan using Bayesian Model, 1990–2035. (A) Policy implement by 2030 and (B) Policy implement by 2035.

The Bayesian analysis indicated that if the Japanese government continues its existing cash benefit policy, which is 0.74 percent of GDP from 2023 to 2035, the projected TFR will be 1.25 (95% CrI: 1.19–1.30) in 2025, 1.24 (1.14–1.35) in 2030, and 1.25 (1.06–1.46) in 2035 (Fig. 4, appendix Supplemental Table S8, p.24). Our model estimates that with Japan’s current cash benefit policies, the probability of achieving a TFR above 1.3 is 12.2% by 2030 and 28.9% by 2035. Conversely, if Japan increases its cash benefits to levels comparable to those in Australia, Hungary, and France by 2030, the probability of achieving a sustained increase in the fertility rate—defined as a TFR rising above its lowest recorded point (1.26 in 2022) and trending toward 1.3 or beyond—will significantly increase to 79.3%, 70.3%, and 69.0%, respectively (Fig. 4A, appendix Supplemental Table S8, p.24). Cash benefits in Japan, if raised to those in Australia, Hungary, and France by 2035, are forecast to reverse the decline of fertility by 72.2%, 75.9%, and 59.6%, respectively (Fig. 4B, appendix Supplemental Table S8, p.25). A sensitivity analysis excluding marriage rate as a covariate indicated slightly higher uncertainty in TFR projections (Supplemental Fig. S5, p.26). However, the overall trend remained consistent, reinforcing the robustness of our model. Given the strong link between marriage and childbirth in Asian societies, we retained marriage rate in the final model while acknowledging its potential confounding effect.

### Sensitivity analysis and model diagnostic

After testing the model’s validity, the posterior predictive check yielded p-values around 0.5 (appendix, Supplemental Fig. S6, p.27), which suggests that the model accurately replicates the observed data, indicating its suitability and reliability for the data analysis. This result validates the model’s accuracy and its potential for reliable future projections. We performed a sensitivity analysis by modifying the priors for the hyperparameters. Altering the prior distributions from a half-Cauchy to gamma distribution did not significantly impact the results (appendix, Supplemental Fig. S7 and Table S9, p. 28–29). In terms of model diagnostics, the potential scale reduction factor (PSRF) values were evaluated, suggesting that both the point estimate and the upper limit of the PSRF for the scenario-based models approached 1 (appendix Supplemental Table S10, p. 30), indicating model convergence and reliability.

### Model validation

The Leave-One-Out Cross-Validation (LOO-CV) analysis compared predictive performance (LOO-LPD) across six countries for 2030 and 2035 scenarios (appendix Supplemental Table S11, p31; Supplemental Fig. S8, p32). Overall, LOO-LPD scores improved from 2030 to 2035 for all countries, indicating better predictive performance or scenario assumptions over time. The LOO-CV analysis showed improved predictive performance (LOO-LPD) across all scenarios from 2030 to 2035. The LOO-CV analysis demonstrated improvements in predictive performance (LOO-LPD) across all scenarios from 2030 to 2035. Australia and France achieved the most significant gains, highlighting successful enhancements in model assumptions and data inputs. Japan consistently ranked the highest, showcasing excellent predictive accuracy, while Hungary maintained stable performance, reflecting reliable but steady results. Germany and Greece exhibited moderate progress, demonstrating consistent improvements over time. Overall, the results emphasize positive advancements across all scenarios, with Japan setting a high standard for reliability, Australia making remarkable strides, and France and Greece showing strong potential for future growth.

## Discussion

In this study, we have identified several key findings. First, our systematic review indicated that among various fertility policies, cash benefits and childcare coverage stand out as the most effective to enhance fertility rates. While tax refunds and unpaid maternity leave also show significant impacts, these areas warrant further research to understand their full impact. Second, we found that under the with Japan’s existing cash benefit policies, there is only a 17% likelihood of reversing the declining fertility rates by 2030. However, should Japan increase its cash benefits to align with those of France, Hungary, and Australia by 2030 or 2035, the probability of reversing the fertility decline would surge to over 75%. Finally, by integrating insights from systematic reviews with secondary data analysis process, we have managed to forecast future fertility trends in Japan. This comprehensive approach provides valuable insights for policymakers, offering a data-driven basis, rather than anecdotes, for potential adjustments in fertility-related policies to address the pressing demographic challenges in Japan.

Our findings suggest that policies such as birth payments, various allowances (monthly, quarterly, or annually), paid maternity leave, childcare coverage, paid parental leave, and tax exemptions are particularly effective in enhancing fertility rates. However, the impact of cash benefits varies across regions and socio-economic groups. While high-income countries often observe positive effects on birth rates, studies like Yamaguchi (2019) highlight limited effects in Japan, where cash benefits are more effective in supporting maternal employment. This underscores the importance of cultural and structural factors—such as societal norms, gender roles, and the availability of childcare and parental leave—in shaping policy success.^39^ Hart et al. (2023) also emphasize that pro-natalist policies can have unequal impacts, with parental leave often benefiting higher-income families and childcare reducing inequalities.^40^ Therefore, while increasing cash benefits can improve fertility, it also risks exacerbating disparities, necessitating comprehensive, equitable policies that integrate financial incentives with broader structural support tailored to Japan’s demographic challenges. Moreover, evidence suggests that simultaneous implementation of multiple, contextually-adapted fertility policies can significantly increase country’s fertility rate.^9^^,^^10^^,^^41^ Although our simulations focus on the demographic impacts of enhanced cash benefits, it is important to acknowledge the associated fiscal costs. Studies like Bick (2016) indicate that funding policies through income tax increases may offset benefits by reducing disposable income, highlighting the need for sustainable financing strategies.^42^ For instance, Germany, which had one of the lowest TFR among the OECD countries in 1995 at 1.23, experienced a steady increase in TFR over the years, reaching 1.59 in 2021. This rise in fertility can be linked to several policy changes, including the announcement of universal childcare coverage in 2005 and the reform of its 1986 childcare policy in January 2007, which provided financial support for parents. These measures illustrate how comprehensive and coordinated policy efforts can effectively address and potentially reverse trends in declining fertility rates. In our study, we also observed a slight reduction in cash benefits and a concurrent incremental rise in service benefits in Germany during 2006–2007. This policy adjustment, coupled with the simultaneous implementation of multiple policies, likely contributed to hold back the declining fertility in Germany.

Conversely, our study found that France experienced sharp increase of cash benefits and a slight reduction in service benefits during 2006–2007, which corresponded with a steady rise of TFR from 1.77 in 1990 to 1.80 in 2022. The increasing trend in France’s fertility could be attributable to the simultaneous implementation of various family-oriented policies, such as the childcare allowance introduced in 1980, universal family allowance implemented in 1997, paternity leave of two weeks since 2002, and 16 weeks of maternity leave initiated 1989.^41^ It is important to recognize that the impact of policies on fertility rates extends beyond the provision of financial incentives. Other factors, such as cultural norms, societal attitudes towards family and work, and the overall availability of family-friendly policies^11^^,^^32^, ^33^, ^34^^,^^43^, ^44^, ^45^, ^46^ can also play a crucial role in shaping fertility decisions. These multifaceted factors collectively contribute to the effectiveness of policies aimed at influencing fertility rates, highlighting the need for a comprehensive approach that considers the broader socio-economic and cultural context.

Japan stands at the forefront of demographic challenges, marked by a sustained decline in fertility rates and an escalating aging population. The sharp reduction in the TFR from 1.54 in 1990 to 1.26 between in 2022 reflects a profound societal transformation. This downturn has transitioned Japan into a super aging society, a shrinking workforce, and potential long-term economic challenges, amplifying the urgency for prompt and effective policy measures. Our study’s scenario-based projections for Japan highlight the limited impact of current fertility policies, indicating a continued decline in fertility rates. Our study reveals that, despite a substantial increase in service benefits since 2004, Japan’s cash benefits have seen only marginal growth, maintaining its position as the lowest among OECD countries. Currently, Japan allocates approximately 0.74% of its GDP to family cash benefits, significantly lower than countries like Australia (1.66%), Hungary (1.72%), France (1.50%), Germany (1.15%), and Greece (1.08%). To align with these nations, Japan would need to increase its expenditure by 0.34–0.98 percentage points of GDP, representing rises between 46% and 132% from the current level. Given Japan’s high public debt, exceeding twice the size of its economy, careful consideration is required in selecting funding strategies. Potential approaches include modest increases in the consumption tax, which was raised to 10% in 2019, with discussions ongoing regarding further adjustments to fund social expenditures. A proposed 1–2 percentage point increase could generate additional revenue to partially cover the enhanced family benefits; however, it is crucial to assess the potential impact on household consumption and economic growth, especially considering current inflationary pressures. Alternatively, implementing progressive income tax adjustments could provide additional revenue, though the effects on work incentives and income distribution would need thorough evaluation. Focusing on enhancing labor productivity through investments in digital innovation and support for key industries could also expand fiscal space over time, providing resources for increased social spending without immediate tax hikes. Further studies are essential to evaluate the optimal funding structure, ensuring that economic trade-offs are minimized while achieving the desired increase in family cash benefits.

Our simulation of Japan’s TFR under cash-benefit policies modeled after Western countries, such as France and Germany, explores the potential impact of enhanced financial incentives on fertility rates. This comparative framework is informed by findings from Kim (2023),^31^ who observed a 4.7% increase in South Korea’s TFR following pro-natalist cash transfers, along with unintended consequences such as changes in the sex ratio at birth and infant health disparities. These insights highlight the complex interplay between financial incentives and demographic behaviors, emphasizing the importance of evaluating potential demographic shifts and unintended outcomes when designing pro-natalist policies for Japan.

Japan has already implemented the “Child Future Strategy” in December 2023, a comprehensive plan aimed at supporting children and families. Under this strategy, Prime Minister Fumio Kishida announced proposals to increase child allowances, introduce a new insurance policy for natural childbirth expenses, and eliminate the income threshold for existing allowances, guaranteeing monthly payments for all parents with children through high school. The Child Future Strategy also introduces measures such as tuition waivers for university education, subsidies for parental leave and part-time childcare, and enhanced paternity leave benefits.^47^ By 2025, Japan aims to have 50% of new fathers take paternity leave, with a target of reaching 85% by 2030.^47^ Japan’s unique socio-cultural context, characterized by shifting societal norms, economic dynamics, and the evolving aspirations among the younger population, introduces additional complexity to policy formulation. Navigating the interplay between traditional values and contemporary aspirations is essential, as is tackling issues related to gender roles, work-life balance, and housing affordability, to develop effective and culturally sensitive interventions. Critics of these strategies highlight concerns regarding financial sustainability and potential reductions in work incentives. However, advocates believe that these policy measures are crucial for enhancing families support and promoting equal opportunities for all. Given our comprehensive evidence synthesis and scenario-based projections, Japan needs to scale up its existing cash benefit policies, such as childcare allowances and paid parental leave. Simultaneously, the introduction of new financial incentives, including birth payments and tax benefits, could successfully increase Japan’s fertility rates.

The primary advantage of our study is its capability to provide valuable insight into policies that boost fertility, utilizing a systematic review and secondary data analysis of diverse governmental strategies. Our study’s findings clearly demonstrate that family benefits, including childcare support, cash transfers such as baby bonuses and paid maternity leave, as well as tax exemptions, are among the most impactful fertility-enhancing policies. This valuable information will significantly aid policy makers in Japan in tackling the persistent challenges of declining fertility rates, guiding them to devise and expand effective policy measures. We adhered to the PRISMA 2020 reporting guidelines to ensure transparent and high-quality reporting of our systematic review, enhancing the clarity and reliability of our study (Appendix Supplemental Table S12, pp. 33–34). Additionally, our study employed advanced statistical models, such as Bayesian approaches, to accurately forecast the potential impact of various policies on Japan’s future fertility rates, taking into account various scenario-based cash benefit policies.

However, our study has several limitations, which warrant acknowledgement. Firstly, we were unable to conduct a meta-analysis as there was no standardized estimation process available to establish a direct relationship between public policy and fertility rates. Secondly, our systematic review was predominantly focused on European studies, with limited input from Asian contexts, which may affect the generalizability of our findings. Thirdly, the studies we reviewed did not consistently account for the reform stages of policies, which limited our ability to adjust for temporal effects in our evidence synthesis. Fourth, while this study focuses on the demographic impact of cash benefits, further research is needed to incorporate a detailed cost-benefit analysis, including fiscal sustainability and long-term economic trade-offs. Future studies should explore the dynamic interaction between funding mechanisms and policy outcomes. Fifth limitation of this study is the use of policy variables as a proportion of GDP, which, while enabling cross-country comparisons, does not account for variations in population composition. Alternative metrics, such as per-child or per-young-female ratios, could provide more targeted insights but are constrained by the lack of consistent long-term data. Despite these limitations, GDP-based measures remain a practical and widely accepted approach for global analyses. Future research should focus on developing more refined datasets to enhance policy impact assessments. Six, this study categorizes various cash benefit policies under a broad umbrella, which may overlook critical distinctions arising from specific conditions such as means-testing or parental employment requirements. These factors can substantially affect the outcomes of such policies, as noted by Guner et al. (2020).^48^ Future research should aim to disaggregate cash benefit policies to examine the distinct effects of each type, thereby providing more tailored and effective policy recommendations. Seven, we did not conduct methodological appraisal of the studies. However, all of the studies used robust methodologies and were published in peer reviewed journals. Finally, the absence of data restricted us from conducting fertility projections stratified by parity, age, income, or residence in our secondary data analysis. These limitations should be carefully considered when interpreting the findings of our study.

### Conclusion and policy implications

In conclusion, our research findings indicate that cash benefits play a significant role in increasing fertility rates in high-income countries. Our study suggests a significant likelihood—over 75%—that Japan could reverse its fertility rate decline if it aligns its cash benefit policy with those of France, Hungary, and Australia by 2030 or 2035. However, Japan’s investment in cash transfers is significantly low compared to the majority of OCED countries. While cash benefits, including parental leave, are effective, they should not be considered as the sole remedy. Enhanced cash benefits pose a challenge for policymakers, as funding mechanisms like tax increases or budget shifts can impact their effectiveness. While cash incentives may boost fertility rates, fiscal measures could reduce household disposable income, potentially negating these effects. Sustainable approaches, such as progressive tax reforms or resource reallocation, are essential to achieving demographic goals without compromising economic stability. A broader spectrum of strategies, including enhanced childcare services, and tax exemptions or refunds, should also be considered to promote higher fertility rates. Therefore, we recommend a recalibration of Japan’s cash benefit policies, urging the adoption of a comprehensive policy framework that tackles various factors influencing fertility rates. An increase in cash benefits, coupled with sustained service benefits, is imperative to address the needs of individuals and families in a holistic manner. Through such proactive policy measure, Japan can embark on a path to reverse its fertility rate decline and secure a demographically sustainable future.

## Contributors

KS and MMR conceived the study and contributed to its statistical analysis. MMR drafted the first draft. MMR, HS, and SS finalized the manuscript draft. MS and MAA were used to verify the consistency of the analysis and the study. KS reviewed the manuscript critically. The final manuscript was reviewed and approved by all authors.

## Data sharing statement

The data follows the Guidelines for Accurate and Transparent health Estimates reporting. Requests for data-sharing will be considered by the corresponding author.

## Declaration of interests

We declare no competing interests.
