# Supplementary material for: Reversing fertility decline in Japan with foreign pro-natalist policies, 1990–2035: a systematic review and secondary data analysis
Source: Lancet Reg Health West Pac. 2025 Jun 14;59:101596. doi: 10.1016/j.lanwpc.2025.101596 (PMC12206145; doi:10.1016/j.lanwpc.2025.101596)
Supplement: Supplemental Figs. S1–S8 and Tables S1–S12 [file mmc1.docx]

**Supplemental appendix**

**Reversing fertility decline in Japan** **with foreign** **pro-natalist policies, 1990-2035: a systematic review and secondary data analysis**

Md. Miznaur Rahman, PhD^1,2^; Haruka Sakamoto, PhD^2,3^; Sabera Sultana, PhD^2^; Miho Sassa, PhD^4^; Md Ashraful Alam, PhD^5^; Kenji Shibuya, DrPH^2^

^1^Research Center for Health Policy and Economics, Hitotsubashi University, Tokyo, Japan

^2^Tokyo Foundation for Policy Research, Tokyo, Japan

^3^Department of International Health and Tropical Diseases, Tokyo Women’s Medical University, Japan

^4^Department of Global Health Policy, Graduate School of Medicine, The University of Tokyo, Japan

^5^Department of Computational Diagnostic Radiology and Preventive Medicine, The University of Tokyo Hospital, Japan

**eMethod1: systematic review**

A systematic review was conducted for studies using fertility, policy as the key explanatory variable, and experimental methods. Our search strategies and searched records are detailed in this appendix (Tables S1-S3). The search strategies and searched records were designed to avoid bias toward the near and known. Several pre-set criteria were used for the study population (P), type of policy/intervention (I), comparison groups (C), outcome (O), and study design (S). We followed PICOS instructions regarding database search and selection The inclusion criteria for this study include women and men of childbearing age living in upper middle income and high income countries, as well as interventions implemented at the national, regional, or local level which affect fertility choices. Comparisons are made between the introduction and revocation of a policy, as well as modifications of a policy and its previous form. Outcomes such as birth rate, total fertility rate, general fertility rate, complete or period fertility rate, intention to birth, parity or birth transition are also considered. The studies used must be experimental, quasi-experiments, pre-post studies, control before and after, interrupted time series (ITS) or repeated cross-sectional studies. Exclusions are made for target specific populations such as teenage pregnancies or minorities, as well as any observational study that does not use the strategies mentioned above for identifying causal relationships.

Two researchers were independently review the titles and abstracts of the studies retrieved during the search to assess whether they meet the inclusion and exclusion criteria. If there are disagreements, a third researcher will be consulted. The full text versions of the selected articles will also be read by two researchers, with disagreements once again resolved by a third researcher if necessary. Finally, two authors will independently review the articles included, assessing the intervention's nature, methodology, sample size, country, policy, outcomes, time period, outcome measure, type of data, and control variables. Any discrepancies will be discussed or a third researcher consulted. The selection of study was performed based on PRISMA flowchart (appendix Figure S1). Two independent authors extracted range of data from the included studies such as author name, publication year, survey year, country, settings, sample size, name of policy, policy description, policy implementation time, name of outcome, definition of outcome, key results, and impact and results directions per policy. All the included policies were classified into three major group: cash benefits, service benefits, and financial support (appendix Table S4) The brief description of the included study is presented in this appendix (Table S5).

**eMethod2: Study quality assessment**

**Observational studies:** We employed a specific checklist to assess the methodological quality of all included cohort studies using the Newcastle–Ottawa Scale (NOS) recommended by Wells and colleagues ^1^. This system is based on a system of stars (*) awarded for each applicable criterion. Three major domain-based approaches were used for evaluating the observational study quality: selection, comparability, and outcome.

*Cohort Study:* The cohort studies used nine criteria: the representativeness of the exposed cohort, the selection of the non-exposed cohort, ascertainment of exposure, outcome of interest not present at start (maximum of four stars); comparability of the cohorts on the basis of study design and analysis (maximum of two stars); and finally, the assessment of the outcome (maximum of three stars). All studies received a score on the basis of these nine criteria, ranging from 0 to 9. Studies were defined as high quality, score ≥ 6; moderate quality, score 4-5; or low quality, score 0-3.

| Study, year | **Selection** | | | |  | **Comparability** | |  | **Outcome** | | | Total Score |
| --- | --- | --- | --- | --- | --- | --- | --- | --- | --- | --- | --- | --- |
|  | 1 | 2 | 3 | 4 |  | 5A | 5B |  | 6 | 7 | 8 |  |
|  | Exposed cohort truly representative | Non-exposed cohort drawn from the same community | Ascertainment of exposure | Outcome of interest not present at start |  | Cohorts comparable on basis of age | Cohorts comparable on other factor(s) |  | Quality of outcome assessment | Follow-up long enough for outcomes to occur | Complete accounting for cohorts |  |
|  |  |  |  |  |  |  |  |  |  |  |  |  |

*Cross-sectional study:* The cross-sectional studies used 10 criteria: the representativeness of the sample, sample size determination, non-response, ascertainment of exposure (maximum of five stars); comparability of the study on the basis of study design and analysis (maximum of two stars); and finally, the assessment of the outcome (maximum of three stars). All studies received a score on the basis of these nine criteria, ranging from 0 to 10. Studies were defined as high quality, score ≥ 6; moderate quality, score 4-5; or low quality, score 0-3.

| Study, year | **Selection (5 points)** | | | |  | **Comparability (2 points)** | |  | **Outcome (3 points)** | | Total Score |
| --- | --- | --- | --- | --- | --- | --- | --- | --- | --- | --- | --- |
|  | 1 | 2 | 3 | 4 |  | 5A | 5B |  | 6 | 7 |  |
|  | Representativeness of the sample (*) | Sample size (*) | Non-respondents (*) | Ascertainment of the exposure (**) |  | Controls for age (*) | Control for any additional factor (*) |  | Assessment of the outcome (**) | Statistical test (*) |  |
|  |  |  |  |  |  |  |  |  |  |  |  |

*Case-control study:* The case-control studies used 9 criteria: definition of case, representativeness of the case, selection of controls, definition of controls (maximum 4 stars); controls for age, controls for any additional factors (maximum of two stars); ascertainment of exposure, ascertainment of case and controls (maximum of two stars). All studies received a score on the basis of these nine criteria, ranging from 0 to 10. Studies were defined as high quality, score ≥ 6; moderate quality, score 4-5; or low quality, score 0-3.

| Study, year | **Selection (4 points)** | | | |  | **Comparability (2 points)** | |  | **Exposure (3 points)** | | | Total Score |
| --- | --- | --- | --- | --- | --- | --- | --- | --- | --- | --- | --- | --- |
|  | 1 | 2 | 3 | 4 |  | 5A | 5B |  | 6 | 7 |  |  |
|  | Definition of case (*) | Representativeness of the case (*) | Selection of controls (*) | Definition of controls (*) |  | Controls for age (*) | Control for any additional factor (*) |  | Ascertainment of exposure (*) | Ascertainment of case and controls (*) |  |  |

*Experimental studies:* Cochrane EPOC tools were used for assessing the risk of bias for quasi-experimental studies such as **c**ontrolled before and after, and interrupted time series ^2^. Risk of bias was evaluated through the following items: confounding bias, detection bias (only in non-experimental studies- two domains), selection bias, attrition bias, reporting bias, other bias.
(1) Confounding bias: Comparability of groups for quasi-experimental studies. For non-experimental studies, was the policy independent of other changes?

(2) Detection bias: Was the shape of the policy effect pre-specified? Was the policy unlikely to affect data collection?

(3) Selection bias: Sample representativeness.

(4) Attrition bias: Describe the completeness of outcome data for each main outcome, including attrition and exclusions from the analysis. - Reporting bias: State how the possibility of selective outcome reporting was examined by the review authors, and what was found.
(5) Other bias: State any important concerns about bias not addressed in the other domains in the tool.

| Study, year | Confounding bias | Detection Bias1 | Detection Bias2 | Selection Bias | Attrition Bias | Reporting Bias | Other Bias | Total Score |
| --- | --- | --- | --- | --- | --- | --- | --- | --- |
|  |  |  |  |  |  |  |  |  |
|  |  |  |  |  |  |  |  |  |

We rated the overall methodological quality of the included studies as being at low, moderate, or high risk of bias. Non-experimental studies without control group and seven domains of bias was used. The articles where there are 5 or more subcategories with low bias are classified as “Low” in the summary risk of bias; the ones with a number of subcategories between 3 and 4 classified as “Moderate”; and the ones with a number of subcategories between 1 and 2 classified as “High”. For quasi-experimental studies with control group and five domains of bias was used. The articles where there are 4 or more subcategories with low bias are classified as “Low” in the summary risk of bias; the ones with 3 subcategories classified as “Moderate”; and the ones with a number of subcategories between 1 and 2 classified as “High”.

For comparison with observational studies and to make a unique score, we change subcategory name from “High risk of bias” to “low-quality study”; “Moderate risk of bias” to “moderate quality study”, and “Low risk of bias” to “high-quality study”.

Table S1: Search history of PubMed

| Search | Query | Results (inception to Sept 11, 2023) | Results (Sept 12, 2023-March 27, 2025) |
| --- | --- | --- | --- |
| #1 | “longitudinal stud*” [tw] OR "interrupted time series analysis"[MeSH Terms] OR “interrupted time series” [tw] OR “controlled before and after” [tw] OR "before and after study" [tw] OR “quasi-experimental” [tw] OR “quasi experimental*” [tw] OR intervention* OR quasiexperiment or *experiment* OR evaluat* OR effect* OR "instrumental variables"[tw] OR "regression discontinuity"[tw] OR "difference in difference"[tw] OR "difference-in-difference"[tw] OR "diff in diff"[tw] | 14,793,052 | 16,094,848 |
| #2 | "parental leave"[MeSH] OR "parental leave"[tw] OR "maternity leave"[tw] OR "paternity leave"[tw] OR "monetary incentives" [tw] OR "cash transfers"[tw] OR "cash allowances"[tw] OR baby bonus[tw] OR “discount” [tw] OR lump-sum [tw] OR "lump sum"[tw] OR “cash-for-care”[tw] OR polic* OR "public policy" [MeSH] OR "public policy" [tw] OR "Population level policy" [tw] OR "financial policy"[tw] OR "fiscal policy"[MeSH] | 852,929 | 29,705 |
| #3 | "birth rate"[MeSH] OR “birth rate”[tw] OR birth-rate* OR fertility-rate* OR "fertility rate"[tw] OR "completed fertility"[tw] OR "parity progression"[tw] OR "total fertility rate" [tw] OR “TFR”[tw] OR "cohort fertility rate"[tw] OR "lifetime fertility"[tw] OR "general fertility rates"[tw] | 27,265 | 156,986 |
| #4 | "developing countries"[MeSH] OR "developing countries"[tw] OR "low income countries"[tw] OR "lower-middle income countries" [tw] | 148,192 | 1168 |
| #5 | **(#1 AND #2 AND #3) NOT #4** | 1,000 |  |
|  | **(#1 AND #2 AND #3) NOT #4 Filters:** from 2023/10/12 - 2025/3/27 | - | 166 |

Table S2: **Search history of Web of Science**

| Search | Query | Results (inception to 2023) | Results (2024 to 2025) |
| --- | --- | --- | --- |
| #1 | TI=(“longitudinal stud*”OR "interrupted time series analysis" OR “interrupted time series” OR “controlled before and after” OR "before and after study" OR “quasi-experimental” OR “quasi experimental*” OR intervention* OR quasiexperiment or *experiment* OR evaluat* OR effect* OR "instrumental variables" OR "regression discontinuity" OR "difference in difference" OR "difference-in-difference" OR "diff in diff") OR TS=(“longitudinal stud*”OR "interrupted time series analysis" OR “interrupted time series” OR “controlled before and after” OR "before and after study" OR “quasi-experimental” OR “quasi experimental*” OR intervention* OR quasiexperiment or *experiment* OR evaluat* OR effect* OR "instrumental variables" OR "regression discontinuity" OR "difference in difference" OR "difference-in-difference" OR "diff in diff") | 25,287,032 | 25,287,032 |
| #2 | TI=("parental leave" OR "parental leave" OR "maternity leave" OR "paternity leave" OR "monetary incentives" OR "cash transfers" OR "cash allowances" OR baby bonus OR “discount” OR lump-sum OR "lump sum" OR “cash-for-care” OR polic* OR "public policy" OR "public policy" OR "Population level policy" OR "financial policy" OR "fiscal policy") OR TS=("parental leave" OR "parental leave" OR "maternity leave" OR "paternity leave" OR "monetary incentives" OR "cash transfers" OR "cash allowances" OR baby bonus OR “discount” OR lump-sum OR "lump sum" OR “cash-for-care” OR polic* OR "public policy" OR "public policy" OR "Population level policy" OR "financial policy" OR "fiscal policy") | 1420163 | 1420163 |
| #3 | TI=("birth rate" OR “birth rate” OR birth-rate* OR fertility-rate* OR "fertility rate" OR "completed fertility" OR "parity progression" OR "total fertility rate" OR “TFR” OR "cohort fertility rate" OR "lifetime fertility" OR "general fertility rates") OR TS=("birth rate" OR “birth rate” OR birth-rate* OR fertility-rate* OR "fertility rate" OR "completed fertility" OR "parity progression" OR "total fertility rate" OR “TFR” OR "cohort fertility rate" OR "lifetime fertility" OR "general fertility rates") | 27,561 | 27,561 |
| #4 | TI=("developing countries” OR "developing countries" OR "low income countries" OR "lower-middle income countries") OR TS=("developing countries" OR "developing countries" OR "low income countries" OR "lower-middle income countries") | 153,690 | 153,690 |
| #5 | (#1 AND #2 AND #3 NOT #4) | 1168 | 1168 |
| #6 | (#1 AND #2 AND #3 NOT #4) **Filters:** from 2024- 2025 | - | 156 |

Table S3: Search history of CINAHL

| Search | Query | September 11, 2023 |
| --- | --- | --- |
| #1 | TI("parental leave" OR "parental leave" OR "maternity leave" OR "paternity leave" OR "monetary incentives" OR "cash transfers" OR "cash allowances" OR baby bonus OR “discount” OR lump-sum OR "lump sum" OR “cash-for-care” OR polic* OR "public policy" OR "public policy" OR "Population level policy" OR "financial policy" OR "fiscal policy") OR AB("parental leave" OR "parental leave" OR "maternity leave" OR "paternity leave" OR "monetary incentives" OR "cash transfers" OR "cash allowances" OR baby bonus OR “discount” OR lump-sum OR "lump sum" OR “cash-for-care” OR polic* OR "public policy" OR "public policy" OR "Population level policy" OR "financial policy" OR "fiscal policy") | 11,348,028 |
| #2 | TI("birth rate" OR “birth rate” OR birth-rate* OR fertility-rate* OR "fertility rate" OR "completed fertility" OR "parity progression" OR "total fertility rate" OR “TFR” OR "cohort fertility rate" OR "lifetime fertility" OR "general fertility rates") OR AB("birth rate" OR “birth rate” OR birth-rate* OR fertility-rate* OR "fertility rate" OR "completed fertility" OR "parity progression" OR "total fertility rate" OR “TFR” OR "cohort fertility rate" OR "lifetime fertility" OR "general fertility rates") | 1,024,768 |
| #3 | TI("developing countries” OR "developing countries" OR "low income countries" OR "lower-middle income countries") OR AB("developing countries" OR "developing countries" OR "low income countries" OR "lower-middle income countries") | 78,345 |
| #4 | #1 AND #2 AND #3 | 515 |

Table S4: Family and childcare support public expenditure

| Policy | Policy sub-category | Short description^a^ |
| --- | --- | --- |
| Cash benefits | Income maintenance benefit in the event of a childbirth | Earning-related of flat rate compensations for the loss of earnings due to temporary exit from the labour market in the period of childbirth. |
|  | Birth grant | Lump-sum benefits in case of childbirth. |
|  | Parental leave benefit | Payments made to one parent which interrupted work for early childrearing. |
|  | Family and child allowance | Periodical payments to one parent with dependent children. |
|  | Other cash benefits | Various other lumpsum or periodical payments in order to help families with specific needs, such as lone parent families or families with disabled children. |
| Services benefits | Child day care | Shelter and board provided to pre-school children during the day or part of the day. |
|  | Accommodation | Permanent shelter and board provided to children and families, such as in nursing homes and foster families. |
|  | Home help | Services provided at home to children and/or to persons who care for them. |
| Tax-breaks | Tax-based financial support for families | In this category are tax exemptions (e.g., income from child benefits that isn't included in the tax base); child tax allowances (amounts deducted from gross income that do not become taxable income); and child tax credits (amounts deducted from the income tax liability). The cash payment for any excess of the child tax credit over the liability is recorded under cash transfers above (the same applies to cash payments for child tax credits as a general rule). |

^a^Note: Public spending on family benefits, including financial support, includes financial support. In addition to health and housing, other social policy areas also assist families, but not exclusively, so they are not included in this indicator.Public spending on family benefits can be broadly divided into three categories:

- *Child-related cash transfers (cash benefits)* to families with children, including child allowances, whose levels differ with the child's age, and sometimes are income-tested; public income support payments during parental leave and income support to soleparent families.
- *Services for families (benefits in kind)* with children (direct financing and subsidizing childcare and early education providers), public childcare support through earmarked payments to parents, assistance for young people and residential facilities, and services for families.
- *Family financial support is provided by the tax system*, including tax exemptions (e.g. income from child benefits not included in the tax base); child tax allowances, which deduct amounts from gross income and are not included in taxable income, and child tax credits, which are deducted from taxes. This indicator is broken down by cash benefits and benefits in king and is measured in percentage of GDP.

^a^OECD (2023), Family benefits public spending (indicator). doi: 10.1787/8e8b3273-en (Accessed on 17 December 2023)

**e-method 2: Predictor variables preparation**

Data relating to the mean age at first birth, maternal employment rates, marriage rates, and family cash benefits spending as a percentage of GDP from 1990 to 2022 was extracted from the OECD database. GDP per capita from 1990 to 2027 was collected from IMF database. To account for the years 2028 to 2035, it was assumed that GDP per capita would remain the same as the previous years. Other covariates, especially mean age at first birth, marriage rate, and employment rate, were predicted for the years 2023 to 2035 using a Bayesian regression model, where the year was considered as the main covariate. A detailed OECD database can be found at this link: http://www.oecd.org/els/family/database.htm, while an IMF profile on GDP per capita can be found here: <http://www.imf.org/external/datamapper/profile/JPN>. The proportion of the population aged 0–14, expressed as a percentage of the total population, was obtained from the United Nations (UN) World Population Prospects (WPP). Here WPP data sources: https://population.un.org/wpp/.

**Table S5: Japan’s cash benefits policy by 2030 or 2035**

| Year | Japan  (current  cash benefit)^a^ | Japan adopts foreign cash benefits policy from 2024 to 2030^b^ or 2035^c^ | | | | | | | | | |
| --- | --- | --- | --- | --- | --- | --- | --- | --- | --- | --- | --- |
|  |  | Australia | | France | | Germany | | Greece | | Hungary | |
|  |  | 2030^b^ | 2035^c^ | 2030 | 2035 | 2030 | 2035 | 2030 | 2035 | 2030 | 2035 |
| 2023 | 0.7424641 | 0.86 | 0.82 | 0.84 | 0.80 | 0.79 | 0.77 | 0.78 | 0.77 | 0.86 | 0.82 |
| 2024 | 0.7424641 | 0.97 | 0.88 | 0.93 | 0.86 | 0.84 | 0.81 | 0.83 | 0.79 | 0.99 | 0.89 |
| 2025 | 0.7424641 | 1.09 | 0.95 | 1.03 | 0.92 | 0.90 | 0.84 | 0.87 | 0.82 | 1.11 | 0.97 |
| 2026 | 0.7424641 | 1.20 | 1.02 | 1.12 | 0.98 | 0.95 | 0.87 | 0.91 | 0.85 | 1.23 | 1.04 |
| 2027 | 0.7424641 | 1.32 | 1.10 | 1.22 | 1.03 | 1.00 | 0.90 | 0.95 | 0.87 | 1.35 | 1.12 |
| 2028 | 0.7424641 | 1.43 | 1.17 | 1.31 | 1.09 | 1.05 | 0.93 | 1.00 | 0.90 | 1.48 | 1.19 |
| 2029 | 0.7424641 | 1.55 | 1.24 | 1.41 | 1.15 | 1.10 | 0.96 | 1.04 | 0.92 | 1.60 | 1.27 |
| 2030 | 0.7424641 | 1.66 | 1.31 | 1.50 | 1.21 | 1.15 | 0.99 | 1.08 | 0.95 | 1.72 | 1.34 |
| 2031 | 0.7424641 | 1.66 | 1.38 | 1.50 | 1.27 | 1.15 | 1.02 | 1.08 | 0.98 | 1.72 | 1.42 |
| 2032 | 0.7424641 | 1.66 | 1.45 | 1.50 | 1.33 | 1.15 | 1.06 | 1.08 | 1.00 | 1.72 | 1.49 |
| 2033 | 0.7424641 | 1.66 | 1.52 | 1.50 | 1.38 | 1.15 | 1.09 | 1.08 | 1.03 | 1.72 | 1.57 |
| 2034 | 0.7424641 | 1.66 | 1.59 | 1.50 | 1.44 | 1.15 | 1.12 | 1.08 | 1.05 | 1.72 | 1.64 |
| 2035 | 0.7424641 | 1.66 | 1.66 | 1.50 | 1.50 | 1.15 | 1.15 | 1.08 | 1.08 | 1.72 | 1.72 |

^a^Current model: Japan’s cash benefits assume same as 2021 from 2022 to 2035.

^b^ By 2030, Japan aims to increase its current cash benefits in line with its current foreign policies.

^c^By 2035, Japan aims to increase its current cash benefits in line with its current foreign policies.

Note: The green value indicated is Japan's final target. All the values from 2024 to 2029 or 2024 to 2034 have been calculated using the linear interpolation method to reach the final target. General formula for linear interpolation between two points ($(x_{0},y_{0})$ and ($(x_{1},y_{1})$:

$$y=y_{0}+(x-x_{0})\frac{(y_{1}-y_{0})}{(x_{1}-x_{0})}$$

Specifically, if the value at point X is missing, both the last actual assessment before point X and the first actual assessment after point X are used to calculate it. Coordinates for A, B, and X are (atime, avalue), (btime, bvalue), and (xtime, xvalue). Calculate missing value xvalue at time x for X observation using following equation:

$$xvalue=bvalue+[\frac{\left( avalue-bvalue \right)*\left( xvalue-btime \right)}{(atime-btime)}]$$


Figure S1: PRISMA flowchart for study selection

Table S6: Study characteristics of the included studies (n=61)

| **Study** | **Survey year** | **Country** | **Study design** | **Policy name** | **Policy sub category** | **Outcome name** | **Results direction** | Study Quality^a^ |
| --- | --- | --- | --- | --- | --- | --- | --- | --- |
| Acs, 1996^3^ | 1979-1988 | USA | Interrupted time series | Cash benefits (monthly allowance) | Individual assessment: basic monthly allowance (AFDC), incremental benefit for 2nd child. | Birth transition | Positive (insignificant) | High |
| Ang, 2015^4^ | 2000 to 2008 | Canada | Interrupted time series | Cash benefits (paid maternity leave), Tax exemption | Maternity leave cash benefit, payment for higher parity birth | Birth rate | Positive (significant) | High |
| Baizan and Arpino, 2016^5^ | 2004-2009 | 16 European countries | Longitudinal | Cash benefits (monthly allowance, paid parental leave), Childcare coverage | Family allowances, Childcare coverage, Paid parental leaves | Completed fertility rate | Family allowances: overall positive (insignificant);  Childcare coverage: positive (significant)  Paid parental leaves: positive (insignificant) | High |
| Bauernschuster et al., 2016^6^ | 1998-2010 | Germany | Repeated cross-sectional | Childcare coverage | Child care coverage | Birth rate | Positive (significant) | High |
| Baughman and Dickert-Conlin, 2009^7^ | 1990-1999 | USA | Cross-sectional | Tax refund | Tax rebate | Birth rate | Negative (significant) | High |
| Bonoli, 2008^8^ | 2000-2003, 1980-2000 | Switzerland | Repeated cross-sectional | Cash benefits (monthly allowance), Childcare coverage | Monthly allowance, childcare coverage | TFR | Positive (significant) | High |
| Bick A, 2016^9^ | 1983-2005 | Germany | Panel survey | Childcare coverage | Subsidized child care available | TFR | Positive (insignificant) | High |
| Bokun, 2024^10^ | 2010-2018 | Poland | Cross-sectional | Cash benefits (at birth) | Monthly cash transfer under children 18 years | Fertility rate | Positive (significant) | High |
| Chen et al., 2018^11^ | 1996, 2006, 2011 | Australia | Cross-sectional | Cash benefits (at birth) | Payment at birth or baby bonus | TFR | Positive (significant) | High |
| Cha et al., 2023^12^ | 2015-2019 | South Korea | Interrupted time series | ART health insurance coverage | ART health insurance coverage | Birth rate | Positive (significant) | High |
| Chen and Wei, 2022^13^ | 2008-2017 | China | Interrupted time series | China's two child policy |  | Number of births | Positive (significant) | High |
| Chuard and Chuard-Keller, 2021^14^ | 1969-2017 | Switzerland | Time series | Cash benefits (at birth) | Birth allowance | TFR; Crude birth rate | Positive (significant) | High |
| Cowan and Douds, 2022^15^ | 1982-2010 | USA | Interrupted time series | Cash benefits (annual allowance) | Cash transfer | Birth rate | Positive (significant) | High |
| Cygan-Rehm, 2015^16^ | 2007-2012 | Germany | Before After | Cash benefits (paid parental leave) | Composite: leave duration and cash benefit. | Parity | Higher income group: positive (significant).  Lower income group: negative (significant). | High |
| Dinale, 2024^17^ | 2000-2009 | OECD countries | Times series | Cash benefits, tax-based financial supports, services and in-kind benefits | Child-related cash benefits; tax-based financial support for children; services and in-kind benefits | Fertility rates | All outcomes:  Positive (significant) | High |
| Dahl and Loken, 2016^18^ | 1987-1992 | Norway | Longitudinal Study | Cash benefits (paid maternity leave) | Paid parental leave | Number of children | Insignificant | High |
| Drago et al., 2011^19^ | 2001-2006 | Australia | Cohort | Cash benefits (at birth) | Payment at birth or baby bonus | Fertility rate | Positive (significant) | High |
| Duvander et al., 2019^20^ | 1988-1999 (sweden), 1993-2003 (Norway) | Norway, Sweden | Repeated cross-sectional | Cash benefits (paid parental leave) | Paternal leave | Parity progression | Positive (significant) | High |
| Einarsdóttir et al., 2012^21^ | 2001-2008 | Australia | Interrupted time series | Cash benefits (at birth) | Payment at birth or baby bonus | Birth rate | Positive (significant) | High |
| Einarsdóttir, 2023^22^ | 2002-2019 | Iceland | Repeated cross-sectional | Cash benefits (paid parental leave) | Paid parental leave | TFR | Positive (significant) | High |
| Enache, 2013^23^ | 2000-2008 | Multi-country | Time series | Cash benefits (allowance) | all cash benefits, all public services provided to ease child birth and rearing and all other benefits in kind, means or non- means tested. | Crude birth rate | Positive (significant) | High |
| Farréa and González, 2019^24^ | 2005-2013 | Spain | Interrupted time series | Cash benefits (paid parental leave) | Paternal leave | Delay in subsequent birth | Negative (significant). | High |
| Fukai, 2017^25^ | 2000-2010 | Japan | Cross-sectional | Childcare coverage | Child care coverage and service hour | Fertility rate | Positive (significant) | High |
| Gabos et al., 2009^26^ | 1950-2006 | Hungary | Interrupted time series | Cash benefits (at birth, allowance, paid maternity leave), tax exemption | Composite assessment: family allowance, tax relief, childcare fee, payment at birth, monthly maternity allowance, monthly allowance for nonworking mothers;  Pension payment scheme. | Fertility rate | Child-related benefits: positive (significant);  Pension payment scheme: negative (significant) | Moderate |
| Gauthier and Hatzius, 1997^27^ | 1970-1990 | 22 European countries | Repeated cross-sectional | Cash benefits (allowance, paid maternity leave) | Individual assessment: Paid maternity leave, monthly allowance | Fertility rate | Paid maternity leave: insignificant;  family benefits (direct cash): positive (significant) | High |
| Gohmann and Ohsfeldt, 1994^28^ | 1915-1988 | USA | Interrupted time series | Tax exemption | Dependent tax exemption, restricting abortion | TFR | Positive (significant) | Moderate |
| González and Trommlerová, 2023^29^ | 2000-2017 | Spain | Interrupted time series | Cash benefits (at birth) | Cash payment at birth | Birth rate | Implementation of cash transfer policy: positive (significant);  cancellation of cash transfer policy: negative (significant) | High |
| Harknett et al., 2014^30^ | 2004-2005, 2008-2009 | 20 European countries | Repeated cross-sectional | Cash benefits (paid parental leave) | Paid parental leave duration | Having a birth | Effect was nearly zero (insignificant) | High |
| Hart et al., 2022^31^ | 2007-2016 | Norway | Cross-sectional | Cash benefits (paid parental leave) | Parental leave benefit | Fertility rate | Negative (insignificant) | High |
| Hong et al., 2016^32^ | 2005-2010 | South Korea | Cross-sectional | Cash benefits (at birth) | Payment at birth | TFR, crude birth rate | Positive (significant) | High |
| Jeong et al., 2022^33^ | 2014-2018 | South Korea | Cross-sectional | Cash benefits (allowance), Child rearing services | Individual assessment: cash benefit, in-kind benefit, vouchers, services, education and public relations | Fertility rate | Positive (significant) | High |
| Jonsson, 2018^34^ | 1953-1997 | Iceland | Longitudinal Study | Cash benefits (paid parental leave), Childcare coverage | Composite | Fertility rate, parity progression | Positive (significant) | High |
| Kim and Luke, 2020^35^ | 2007-2014 | South Korea | Longitudinal Survey | Cash benefits (paid parental leave) | Paid parental leave benefit | Parity progression | Positive (significant) | High |
| Kim and Parish, 2022^36^ | 1985-2002 | Canada | Quasi-experimental | Cash benefits (at birth) | Payment at birth | Fertility rate | Positive (significant) | High |
| Kim w, 2024^37^ | 2000-2015 | South Korea | Longitudinal Survey | Cash benefits (at birth) | Cash benefits/baby bonuses | TFR | Positive (significant) | High |
| Kim et al., 2024^38^ | 2008-2022 | South Korea | Panel study | Cash benefits (allowance) | A $61/month child allowance per child | Fertility rate | Positive (significant) | High |
| Kim, 2014^39^ | 1991-1996 | Canada | Quasi-experimental | Cash benefits (at birth) | Payment at birth | Completed fertility rate | Negative (significant) | High |
| Lalive and Zweimülle, 2009^40^ | 1987, 1990, 1996 | Austria | Interrupted time series | Cash benefits (paid parental leave) | Parental leave duration | Number of births | Positive (significant) | High |
| Langridge et al., 2010^41^ | 1995-2006 | Australia | Longitudinal survey | Cash benefits (at birth) | Payment at birth or baby bonus | Fertility rate | Positive (significant) | High |
| Lee and Lee, 2014^42^ | 1971-2009 | Japan | Repeated cross-sectional | Childcare coverage | Child care coverage | TFR | Positive (significant) | High |
| Li et al., 2019^43^ | 2014-2017 | China | Before- after study | Universal two-child policy | Two-child policy allowed all couples to have two children, replacing the one-child policy. | Number of births | Positive (significant) | High |
| Lin et al., 2024^44^ | 1990-2010 | China | Quasi-experimental | Universal two-child policy | Two-child policy allowed all couples to have two children, replacing the one-child policy. | Birth rate | Positive (significant) | High |
| Luci-Greulich et a., 2013^45^ | 1982-2007 | 18 OECD Country | Repeated cross-sectional | Cash benefits (allowance), Childcare coverage | Spending on cash benefits per child (%GDP), spending per birth (%GDP), spending on child care service per child (GDP%) | TFR | Positive (significant) | High |
| Malkov, 2018^46^ | 1976-1989 | Russia | Before After | Cash benefits (at birth, paid parental leave) | Composite: payment at birth and payment during matrnity leave | GFR | Positive (significant) | Moderate |
| Milligan, 2005^47^ | 1987-1997 | Canada | Before After | Cash benefits (at birth) | Payment at birth, payment for higher parity birth | TFR | Positive (significant) | Low |
| Neugart and Ohlsson, 2013^48^ | 2004-2007 | Germany | Quasi-experimental | Cash benefits (allowance) | Monthly allowance | Number of births | Positive (significant) | Low |
| Parr and Guest, 2011^49^ | 2011-2008 | Australia | Longitudinal survey | Cash benefits (at birth), Tax refund | Tax rebate, baby bonus (AD 3000-4000), childcare rebate (30% of total cost and maximum AD4000 per child) | Fertility rate | Positive (insignificant) | Low |
| Raute, 2019^50^ | 2003-2012 | Germany | Quasi-experimental | Cash benefits (paid maternity leave) | Maternity leave benefits | Birth rate | Positive (significant) | Moderate |
| Ridao-Cano et al., 2005^51^ | 1948-1997 | USA | Before After | Tax exemption | Tax exemption | Fertility rate | Positive (significant) | Low |
| Risse, 2006^52^ | 2003 | Australia | Cross-sectional | Maternity leave (paid and unpaid) | Maternity leave (paid and unpaid) | Pregnancy rate | PML <25yrs: positive (significant); PML 25<35: positive (insignificant); PML ≥35: negative (insignificant); UPML <25yrs: positive (significant); UPML 25<35: positive (significant); UPML ≥35: negative (insignificant) | High |
| Reich, 2024^53^ | 1998-2012 | Australia | Times series | Cash benefits (at birth) | Payment at birth or baby bonus | TFR | Positive (significant) | High |
| Sinclair et al., 2012^54^ | 1990-2009 | Australia | Longitudinal survey | Cash benefits (at birth) | Payment at birth or baby bonus | Fertility rate | Positive (significant) | High |
| Soares et al., 2021^55^ | 2010 | Brazil | Cohort | Cash benefits (allowance) | Cash transfer | Fertility rate | Positive (p NA) | High |
| Son 2017^56^ | 2001-2014 | South Korea | Quasi-experimental | Cash benefits (at birth) | Payment at birth | Fertility rate | Positive (significant) | Low |
| Speder et al. 2020^57^ | 2001-2012 | Hungary | Longitudinal Study | Cash benefits (allowance, paid parental leave) | Cash benefits, Paid parental leave | 3rd parity | Positive (significant) | Moderate |
| Thyrian et al., 2010^58^ | 2006-2007 | Germany | Before After | Cash benefits (paid parental leave) | Payment on maternity leave reform | Crude birth rate, GFR | Positive (insignificant) | High |
| Yamaguchi S, 2019^59^ | 1993-2011 | Japan | Panel survey | Parental leave | Parental leave (job protection and cash benefits) | Fertility rate | Positive (insignificant) | High |
| Yun et al., 2023^60^ | 2015-2019 | South Korea | Interrupted time series | ART health insurance coverage | ART health insurance coverage | Birth rate | Negative (significant) | High |
| Wesolowski et al., 2018^61^ | 1995-2011 | 33 Industrial countries | Time series | Cash benefits (allowance) | Cash benefit | TFR | Positive (significant) | Moderate |
| Whittington, 1992^62^ | 1979-1983 | USA | Cross-sectional | Tax exemption | Tax exemption | Birth rate | Positive (significant) | High |
| Winegarden and Bracy, 2019^63^ | 1959, 1969, 1979, 1989 | 17 OECD country | Time series | Cash benefits (paid maternity leave) | Maternity leave | GFR | Positive (significant) | Moderate |

ART, Assisted Reproductive Technology; TFR, total fertility rate; GFR, general fertility rate

^a^We assessed the quality of cross-sectional studies using 10 criteria, including sample representativeness, sample size, non-response, and exposure ascertainment (maximum 5 stars); comparability based on study design and analysis (2 stars); and outcome assessment (3 stars), with total scores ranging from 0 to 10. Case-control studies were evaluated using 9 criteria, including case and control definition, selection, and exposure ascertainment, with the same scoring range and classification: high quality (score ≥6), moderate (4–5), and low (0–3). Non-experimental studies without control groups were assessed using seven domains of bias, while quasi-experimental studies with control groups used five domains. Studies with 5 or more low-bias domains (non-experimental) or 4 or more (quasi-experimental) were classified as low risk; 3–4 (non-experimental) or 3 (quasi-experimental) as moderate risk; and 1–2 as high risk. To enable observational-study comparison, we standardized the terminology: “high risk of bias” was labeled “low-quality study,” “moderate risk” as “moderate quality study,” and “low risk” as “high-quality study.” Details of quality assessment for each study are provided in Appendix Table S13-15.

**Table S7: Systematic review summary cash benefits policies (41 studies)**

| **Country**  **(Study)** | **Policy name** | **Financial benefit** | **% Increase** | | | **1 Unit change in policy** | | |
| --- | --- | --- | --- | --- | --- | --- | --- | --- |
|  |  |  | **Fertility** | **Intention of fertility** | **Higher parity fertility** | **Fertility** | **Intention of fertility** | **Higher parity fertility** |
| Australia | ^1^Paid parental leave scheme | Fulltime minimum wage for 18 weeks |  | 16 |  |  |  |  |
|  | ^2^Birth incentive (one time) | A$3,000 per new child | 12.8 |  | 3.8 |  |  |  |
|  | ^3^Combined cash benefit (70 policies) | Payment at birth (A$ 5000 in 2008), tax rebate (A$ 7500 in 2008) etc. |  |  |  | 0.049 |  | 0.037 |
|  | Child care rebate | Up to 30% maximum A$4000 | NS |  |  |  |  |  |
| Canada | ^4,5^ Birth incentive (quarterly) | Total $500 for 1st child, $1000 for 2nd child, $8000 for third child | 4.4-9 |  |  |  |  |  |
|  | ^6^Paid parental leave scheme | Changed from 55 % to 70% of previous annual income for 25 weeks (2006) | 23.5 |  |  |  |  |  |
| Hungary | ^7^Combined cash benefit | Family allowance, tax relief, childcare fee, payment at birth, maternity allowance, monthly allowance for nonworking mothers with 3rd child. |  |  |  | 0.2 |  | 0.32 |
|  | ^7^Payment for pension scheme | Details of pension scheme NA |  |  |  | -0.02 |  |  |
|  | ^8^Allowance for third birth for nonworking women (Monthly) | 33 % of average womens salary up to 8th birthday of 3rd child |  |  |  |  |  | NS |
|  | ^8^Tax relief | Monthly relief for a one-child family 3,000 HUF, for a two-child family 4,000 HUF per child, for three or more children 10,000 HUF per child. |  |  |  |  |  | 1.584 |
|  | ^8^Universal family allowance | Increased tax relief, unchanged allowance for nonworking mother, increased family allowance. |  |  |  |  |  | 1.652 |
| USA | ^9^Tax relief (1915-1997) | Details of taxation NA |  |  |  | 0.20-0.867 |  |  |
|  | ^10^Universal cash transfer | Annual mean payment was $1547 | 6.5 |  |  |  |  |  |
|  | ^11^Tax relief (1990-2001) | For one child $2428, two child $4008, childless $364 (in 2001) |  |  |  | -0.016--0.009 |  |  |
| Spain | ^12^Policy introduction: birth incentive (one time) | Euro 2500 per birth | 4.7 |  |  |  |  |  |
|  | ^12^Policy cancellation: birth incentive (one time) |  | -5.7 |  |  |  |  |  |
| Germany | ^13,14^Maternity benefit (reform 2006) | Before: monthly 300 euro up to 24 month or 450 euro up to 12 months. After reform: monthly 300-1800 euro up to 12 months | NS |  |  |  |  | -0.012--0.035 |
| Russia | ^15^Maternity benefit (reform 1981) | Monthly payment equalling 27% of the average national female monthly salary and payment at birth equalling 38% and 76% of the average national monthly salary for first and second or third births, respectively | 6.2 |  | 27.6 |  |  |  |
| South Korea | Childcare benefit | USD 100–USD 360 monthly |  |  |  |  | NS |  |

**Figure S2:** Correlation between total fertility rate and duration of maternity leave (weeks), 1990 to 2020


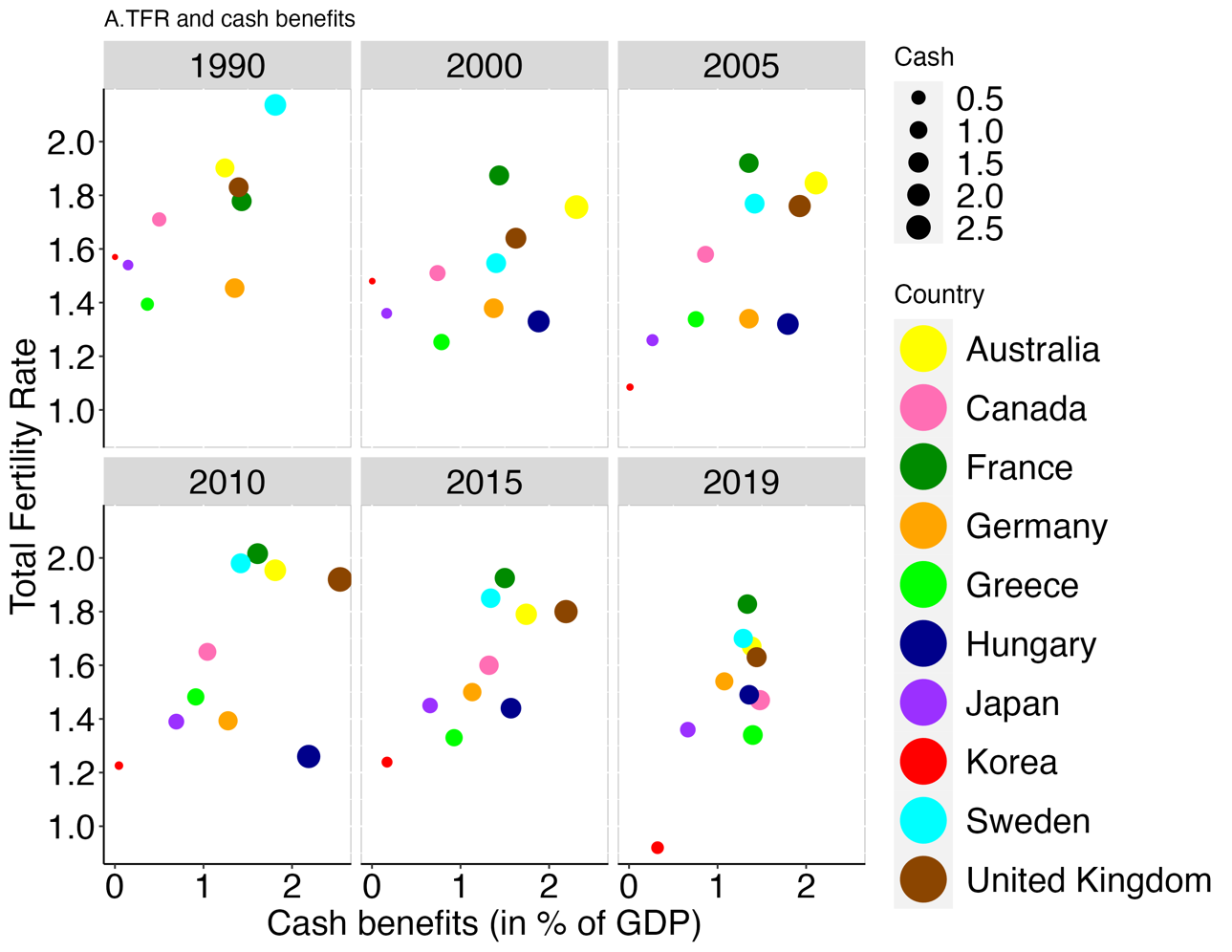


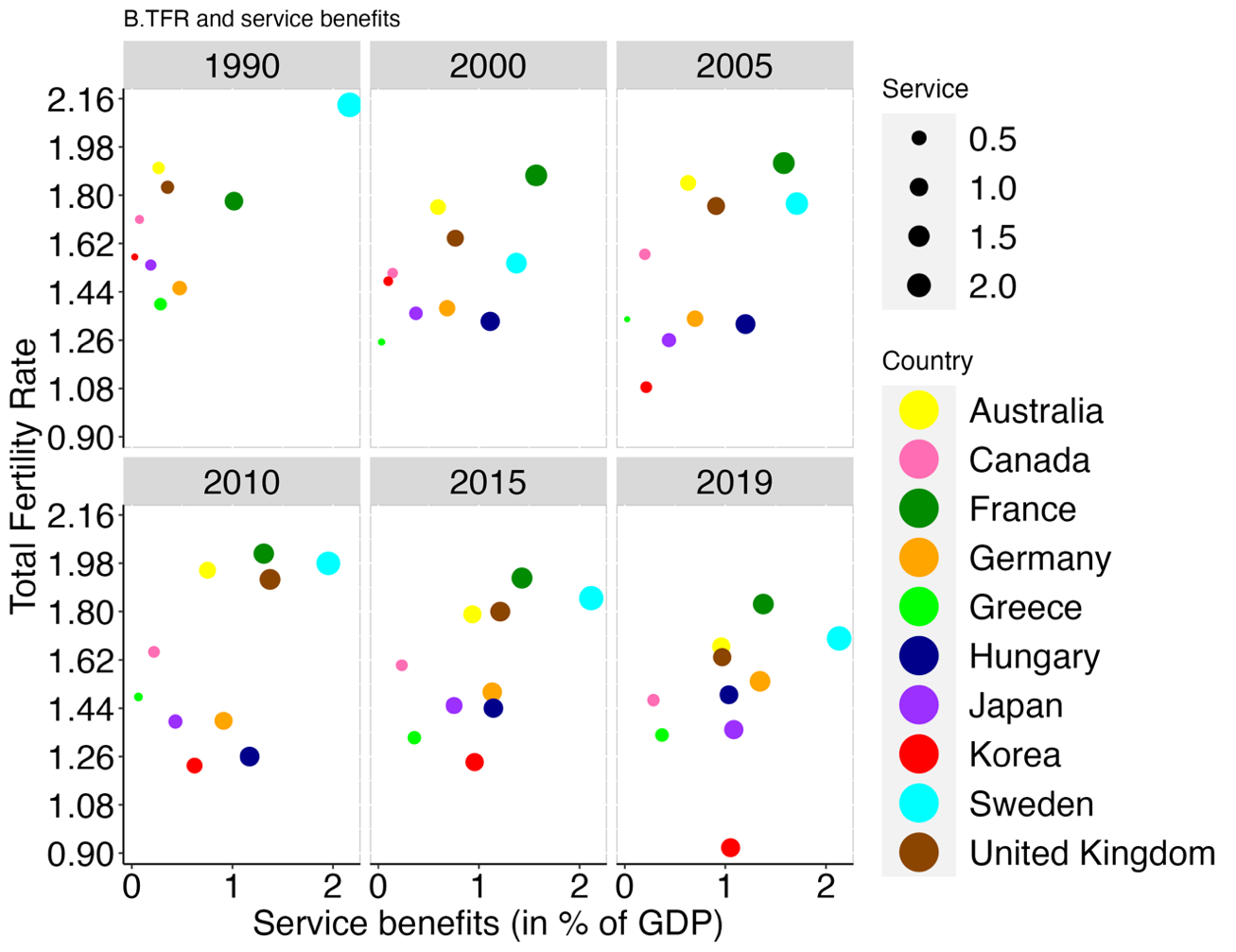


**Figure S3:** Correlation between total fertility rate and family service benefits by selected

countries and years


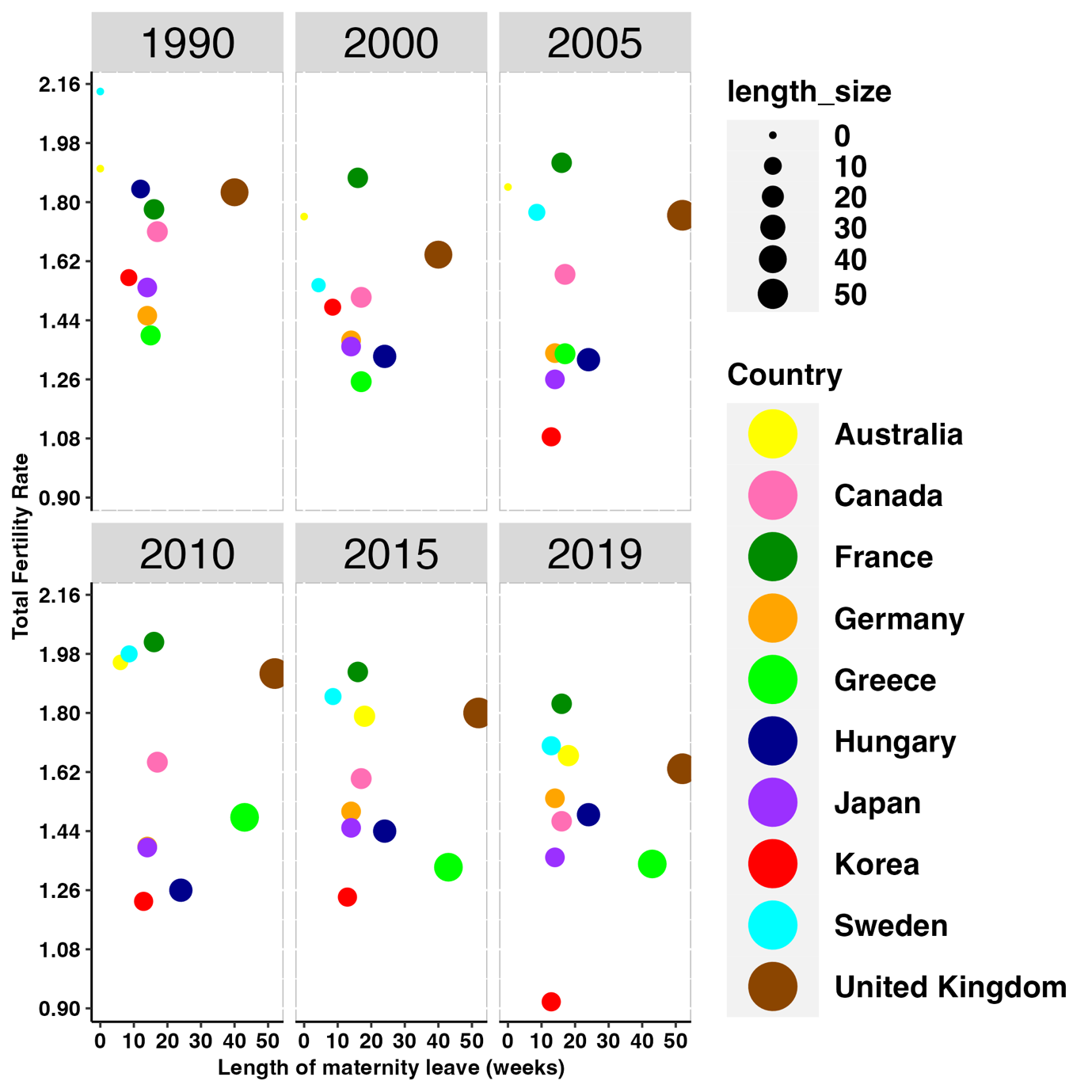


**Figure S4:** Correlation between total fertility rate and length of maternity leave by selected

countries and years

**Table S8:** Scenario-based total fertility rate prediction in Japan

| **Policy** | CASH benefits (% of GDP)^c^ | Predicted TFR for Japan (95% CrI) | | | Prob^y^ to reverse decline TFR=1.30 (threshold value) | | |
| --- | --- | --- | --- | --- | --- | --- | --- |
|  |  | **2025** | **2030** | **2035** | **2030** | **2035** |  |
| **Policy Implemented by 2030^a^** | |  |  |  |  |  |  |
| Japan CBP | 0.70 | 1.25 (1.19–1.3) | 1.24 (1.14–1.35) | 1.25 (1.06–1.46) | 12.2% | 28.9% |  |
| Australia CBP | 0.97 to 1.66 | 1.29 (1.24–1.35) | 1.35 (1.24–1.48) | 1.36 (1.15–1.58) | 79.3% | 68.6% |  |
| Germany CBP | 0.93 to 1.50 | 1.23 (1.19–1.28) | 1.25 (1.15–1.34) | 1.26 (1.08–1.46) | 14.4% | 32.6% |  |
| Greece CBP | 0.84 to 1.15 | 1.26 (1.21–1.32) | 1.28 (1.17–1.39) | 1.27 (1.08–1.49) | 36.3% | 37.6% |  |
| France CBP | 0.83 to 1.08 | 1.28 (1.23–1.33) | 1.33 (1.22–1.44) | 1.35 (1.14–1.57) | 69% | 65.8% |  |
| Hungary CBP | 0.99 to 1.72 | 1.28 (1.23–1.33) | 1.33 (1.22–1.46) | 1.36 (1.15–1.6) | 70.3% | 67.9% |  |
| **Policy Implemented by 2035^b^** | |  |  |  |  |  |  |
| Japan CBP | 0.70 | 1.21 (1.15–1.27) | 1.18 (1.08–1.29) | 1.16 (0.97–1.37) | 1.8% | 8.2% |  |
| Australia CBP | 0.88-1.66 | 1.28 (1.23–1.32) | 1.32 (1.21–1.42) | 1.37 (1.16–1.6) | 61.9% | 72.2% |  |
| Germany CBP | 0.86 to 1.50 | 1.25 (1.2–1.3) | 1.26 (1.16–1.37) | 1.3 (1.1–1.53) | 22.9% | 48.1% |  |
| Greece CBP | 0.81 to 1.15 | 1.25 (1.2–1.3) | 1.25 (1.15–1.36) | 1.27 (1.08–1.5) | 17% | 36.2% |  |
| France CBP | 0.79 to 1.08 | 1.24 (1.18–1.29) | 1.26 (1.15–1.37) | 1.33 (1.11–1.57) | 22.4% | 59.6% |  |
| Hungary CBP | 0.89 to 1.72 | 1.27 (1.21–1.32) | 1.31 (1.19–1.43) | 1.38 (1.17–1.62) | 56.2% | 75.9% |  |

TFR, total fertility rate, CrI, credible interval; CBP cash benefit policy

^a^Implement from 2023 to 2030: The cash benefits will start at the first value in column 2 for 2023 and then linearly increase to reach the foreign policy (last 10 years average) in 2030 and assume the same until 2035.

^b^Implement from 2023 to 2035: The cash benefits will begin in column 2 for 2023and then increase linearly to reach foreign policy (last 10 years average) by 2035.

^c^The details CASH benefits from 2023 to 2035 is presented in Table S7.

**Figure S5:** Scenario-based fertility projection in Japan using Bayesian Model, 1990-2035

Note: fertility projection was done after dropping marriage rate

**Figure S6:** Posterior predictive check plot

**Figure S7:** Scenario-based fertility projection in Japan using Bayesian model based on weekly informative prior, 1990-2035

Table S9: Scenario-based fertility projection in Japan using Bayesian model based on weekly informative prior, 1990-2035

| **Policy** | **Year** | **Predicted TFR (95% CrI)** | |
| --- | --- | --- | --- |
|  |  | **Policy implemented by 2030** | **Policy implemented by 2035** |
| Australia CBP | 2025 | 1.26 (1.21–1.32) | 1.28 (1.23–1.33) |
| Australia CBP | 2030 | 1.3 (1.19–1.43) | 1.32 (1.22–1.42) |
| Australia CBP | 2035 | 1.31 (1.12–1.55) | 1.38 (1.17–1.59) |
| France CBP | 2025 | 1.27 (1.21–1.33) | 1.24 (1.19–1.3) |
| France CBP | 2030 | 1.31 (1.19–1.42) | 1.26 (1.16–1.38) |
| France CBP | 2035 | 1.32 (1.12–1.52) | 1.31 (1.12–1.52) |
| Germany CBP | 2025 | 1.25 (1.19–1.3) | 1.23 (1.18–1.29) |
| Germany CBP | 2030 | 1.26 (1.15–1.37) | 1.23 (1.13–1.34) |
| Germany CBP | 2035 | 1.26 (1.06–1.43) | 1.24 (1.06–1.47) |
| Greece CBP | 2025 | 1.25 (1.2–1.3) | 1.25 (1.19–1.3) |
| Greece CBP | 2030 | 1.27 (1.16–1.38) | 1.25 (1.15–1.37) |
| Greece CBP | 2035 | 1.28 (1.09–1.49) | 1.28 (1.09–1.49) |
| Hungary CBP | 2025 | 1.26 (1.21–1.31) | 1.26 (1.21–1.31) |
| Hungary CBP | 2030 | 1.29 (1.19–1.41) | 1.29 (1.2–1.39) |
| Hungary CBP | 2035 | 1.29 (1.12–1.5) | 1.34 (1.18–1.53) |
| Japan CBP | 2025 | 1.23 (1.18–1.28) | 1.22 (1.17–1.28) |
| Japan CBP | 2030 | 1.22 (1.12–1.32) | 1.2 (1.1–1.31) |
| Japan CBP | 2035 | 1.24 (1.06–1.43) | 1.2 (1.03–1.42) |

CrI, credible interval; CBP, cash benefit policy

**Table S10:** Estimate of Gelman Rubin Potential scale reduction factors (PSRF) for different scenario-based model

| **Year** | Potential scale reduction factors (Point estimate; Upper C.I) | | | | | |
| --- | --- | --- | --- | --- | --- | --- |
|  | Australian CBP | France CBP | German CBP | Greece CBP | Hungary CBP | Japan CBP |
| **1990** | (1.001;1.0042) | (1.0023;1.0074) | (1.0009;1.0021) | (1.0004;1.0024) | (1.002;1.0059) | (1.0013;1.0047) |
| **1995** | (1.0005;1.0028) | (1.0021;1.0067) | (1.001;1.0026) | (1.0004;1.0021) | (1.002;1.0058) | (1.0012;1.0046) |
| **2000** | (1.0005;1.0028) | (1.0021;1.0067) | (1.001;1.0026) | (1.0004;1.0021) | (1.002;1.0058) | (1.0012;1.0046) |
| **2005** | (1.0005;1.0028) | (1.0021;1.0067) | (1.001;1.0026) | (1.0004;1.0021) | (1.002;1.0058) | (1.0012;1.0046) |
| **2010** | (1.0005;1.0028) | (1.0021;1.0067) | (1.001;1.0026) | (1.0004;1.0021) | (1.002;1.0058) | (1.0012;1.0046) |
| **2015** | (1.0005;1.0028) | (1.0021;1.0067) | (1.001;1.0026) | (1.0004;1.0021) | (1.002;1.0058) | (1.0012;1.0046) |
| **2020** | (1.0005;1.0028) | (1.0021;1.0067) | (1.001;1.0026) | (1.0004;1.0021) | (1.002;1.0058) | (1.0012;1.0046) |
| **2025** | (1.0005;1.0028) | (1.0021;1.0067) | (1.001;1.0026) | (1.0004;1.0021) | (1.002;1.0058) | (1.0012;1.0046) |
| **2030** | (1.0005;1.0028) | (1.0021;1.0067) | (1.001;1.0026) | (1.0004;1.0021) | (1.002;1.0058) | (1.0012;1.0046) |
| **2035** | (1.0005;1.0028) | (1.0021;1.0067) | (1.001;1.0026) | (1.0004;1.0021) | (1.002;1.0058) | (1.0012;1.0046) |

CBP, cash benefit policy

Table S11: Changes Between 2030 and 2035 in **Leave-One-Out Cross-Validation (LOO-CV)**

Results

| **Scenario** | **LOO-LPD** | **Rank** |
| --- | --- | --- |
| **Policy Implemented by 2030** |  |  |
| Japan CBP | -55867.3315435905 | 1 |
| Australia CBP | -91553.9255270399 | 5 |
| Germany CBP | -77109.6727619811 | 3 |
| Greece CBP | -72182.9858315451 | 2 |
| France CBP | -87226.1595648819 | 4 |
| Hungary CBP | -94298.6340659955 | 6 |
| **Policy Implemented by 2035** |  |  |
| Japan CBP | -55076.3109862491 | 1 |
| Australia CBP | -84888.9526153188 | 6 |
| Germany CBP | -71636.1569959071 | 3 |
| Greece CBP | -67404.702903287 | 2 |
| France CBP | -81580.0931958107 | 4 |
| Hungary CBP | -84474.8966761449 | 5 |

**LOO-LPD, LOO Log Predictive Density**

Figure S8: Predictive performance (LOO-LPD) across all scenarios from 2030 to 2035

**LOO-LPD, LOO Log Predictive Density**

Table S12: PRISMA check list

| **Section and Topic** | **Item #** | **Checklist item** | **Location where item is reported** |
| --- | --- | --- | --- |
| **TITLE** | | |  |
| Title | 1 | Identify the report as a systematic review. | title |
| **ABSTRACT** | | |  |
| Abstract | 2 | See the PRISMA 2020 for Abstracts checklist. | abstract |
| **INTRODUCTION** | | |  |
| Rationale | 3 | Describe the rationale for the review in the context of existing knowledge. | p.3 |
| Objectives | 4 | Provide an explicit statement of the objective(s) or question(s) the review addresses. | P4 |
| **METHODS** | | |  |
| Eligibility criteria | 5 | Specify the inclusion and exclusion criteria for the review and how studies were grouped for the syntheses. | Appendix p.2 |
| Information sources | 6 | Specify all databases, registers, websites, organisations, reference lists and other sources searched or consulted to identify studies. Specify the date when each source was last searched or consulted. | p.4 |
| Search strategy | 7 | Present the full search strategies for all databases, registers and websites, including any filters and limits used. | Appendix p.3-5 |
| Selection process | 8 | Specify the methods used to decide whether a study met the inclusion criteria of the review, including how many reviewers screened each record and each report retrieved, whether they worked independently, and if applicable, details of automation tools used in the process. | Appendix p.2 |
| Data collection process | 9 | Specify the methods used to collect data from reports, including how many reviewers collected data from each report, whether they worked independently, any processes for obtaining or confirming data from study investigators, and if applicable, details of automation tools used in the process. | Appendix p.2; main text, p.4 |
| Data items | 10a | List and define all outcomes for which data were sought. Specify whether all results that were compatible with each outcome domain in each study were sought (e.g. for all measures, time points, analyses), and if not, the methods used to decide which results to collect. | main text, p.4 |
|  | 10b | List and define all other variables for which data were sought (e.g. participant and intervention characteristics, funding sources). Describe any assumptions made about any missing or unclear information. | main text, p.4-5 |
| Study risk of bias assessment | 11 | Specify the methods used to assess risk of bias in the included studies, including details of the tool(s) used, how many reviewers assessed each study and whether they worked independently, and if applicable, details of automation tools used in the process. | NA |
| Effect measures | 12 | Specify for each outcome the effect measure(s) (e.g. risk ratio, mean difference) used in the synthesis or presentation of results. | NA |
| Synthesis methods | 13a | Describe the processes used to decide which studies were eligible for each synthesis (e.g. tabulating the study intervention characteristics and comparing against the planned groups for each synthesis (item #5)). | NA |
|  | 13b | Describe any methods required to prepare the data for presentation or synthesis, such as handling of missing summary statistics, or data conversions. | NA |
|  | 13c | Describe any methods used to tabulate or visually display results of individual studies and syntheses. | NA |
|  | 13d | Describe any methods used to synthesize results and provide a rationale for the choice(s). If meta-analysis was performed, describe the model(s), method(s) to identify the presence and extent of statistical heterogeneity, and software package(s) used. | NA |
|  | 13e | Describe any methods used to explore possible causes of heterogeneity among study results (e.g. subgroup analysis, meta-regression). | NA |
|  | 13f | Describe any sensitivity analyses conducted to assess robustness of the synthesized results. | NA |
| Reporting bias assessment | 14 | Describe any methods used to assess risk of bias due to missing results in a synthesis (arising from reporting biases). | NA |
| Certainty assessment | 15 | Describe any methods used to assess certainty (or confidence) in the body of evidence for an outcome. | NA |
| **RESULTS** | | |  |
| Study selection | 16a | Describe the results of the search and selection process, from the number of records identified in the search to the number of studies included in the review, ideally using a flow diagram. | Text, p.7; appendix Figure S1 |
|  | 16b | Cite studies that might appear to meet the inclusion criteria, but which were excluded, and explain why they were excluded. | NA |
| Study characteristics | 17 | Cite each included study and present its characteristics. | Appendix Table S6 |
| Risk of bias in studies | 18 | Present assessments of risk of bias for each included study. | NA |
| Results of individual studies | 19 | For all outcomes, present, for each study: (a) summary statistics for each group (where appropriate) and (b) an effect estimate and its precision (e.g. confidence/credible interval), ideally using structured tables or plots. | NA |
| Results of syntheses | 20a | For each synthesis, briefly summarise the characteristics and risk of bias among contributing studies. | Main Table 1-2; Appendix Table S6 |
|  | 20b | Present results of all statistical syntheses conducted. If meta-analysis was done, present for each the summary estimate and its precision (e.g. confidence/credible interval) and measures of statistical heterogeneity. If comparing groups, describe the direction of the effect. | NA |
|  | 20c | Present results of all investigations of possible causes of heterogeneity among study results. | NA |
|  | 20d | Present results of all sensitivity analyses conducted to assess the robustness of the synthesized results. | NA |
| Reporting biases | 21 | Present assessments of risk of bias due to missing results (arising from reporting biases) for each synthesis assessed. | NA |
| Certainty of evidence | 22 | Present assessments of certainty (or confidence) in the body of evidence for each outcome assessed. | NA |
| **DISCUSSION** | | |  |
| Discussion | 23a | Provide a general interpretation of the results in the context of other evidence. | Main text p.10-11 |
|  | 23b | Discuss any limitations of the evidence included in the review. | Main text p.13 |
|  | 23c | Discuss any limitations of the review processes used. | Main text p.13 |
|  | 23d | Discuss implications of the results for practice, policy, and future research. | Main text p.13-14 |
| **OTHER INFORMATION** | | |  |
| Registration and protocol | 24a | Provide registration information for the review, including register name and registration number, or state that the review was not registered. | NA |
|  | 24b | Indicate where the review protocol can be accessed, or state that a protocol was not prepared. | NA |
|  | 24c | Describe and explain any amendments to information provided at registration or in the protocol. | NA |
| Support | 25 | Describe sources of financial or non-financial support for the review, and the role of the funders or sponsors in the review. | Main text p.14 |
| Competing interests | 26 | Declare any competing interests of review authors. | Main text p.14 |
| Availability of data, code and other materials | 27 | Report which of the following are publicly available and where they can be found: template data collection forms; data extracted from included studies; data used for all analyses; analytic code; any other materials used in the review. | Main text p.14 |

*From:*  Page MJ, McKenzie JE, Bossuyt PM, Boutron I, Hoffmann TC, Mulrow CD, et al. The PRISMA 2020 statement: an updated guideline for reporting systematic reviews. BMJ 2021;372:n71. doi: 10.1136/bmj.n71. This work is licensed under CC BY 4.0. To view a copy of this license, visit <https://creativecommons.org/licenses/by/4.0/>

Table S13: Quality assessment of cohort study

| Study, year | **Selection** | | | |  | **Comparability** | |  | **Outcome** | | | Total Score |
| --- | --- | --- | --- | --- | --- | --- | --- | --- | --- | --- | --- | --- |
|  | 1 | 2 | 3 | 4 |  | 5A | 5B |  | 6 | 7 | 8 |  |
|  | Exposed cohort truly representative | Non-exposed cohort drawn from the same community | Ascertainment of exposure | Outcome of interest not present at start |  | Cohorts comparable on basis of age | Cohorts comparable on other factor(s) |  | Quality of outcome assessment | Follow-up long enough for outcomes to occur | Complete accounting for cohorts |  |
| Drago et al., 2011^19^ | * | * | * | * |  | * | * |  | * | * | * | 9 |
| Soares et al., 2021^55^ | * | * | * |  |  | * | * |  | * |  | * | 7 |

Note: The cohort studies used nine criteria: the representativeness of the exposed cohort, the selection of the non-exposed cohort, ascertainment of exposure, outcome of interest not present at start (maximum of four stars); comparability of the cohorts on the basis of study design and analysis (maximum of two stars); and finally, the assessment of the outcome (maximum of three stars). All studies received a score on the basis of these nine criteria, ranging from 0 to 9. Studies were defined as high quality, score ≥ 6; moderate quality, score 4-5; or low quality, score 0-3.

Table S14: Quality assessment of cross-sectional study

| Study, year | **Selection (5 points)** | | | |  | **Comparability (2 points)** | |  | **Outcome (3 points)** | | Total Score |
| --- | --- | --- | --- | --- | --- | --- | --- | --- | --- | --- | --- |
|  | 1 | 2 | 3 | 4 |  | 5A | 5B |  | 6 | 7 |  |
|  | Representativeness of the sample (*) | Sample size (*) | Non-respondents (*) | Ascertainment of the exposure (**) |  | Controls for age (*) | Control for any additional factor (*) |  | Assessment of the outcome (**) | Statistical test (*) |  |
| Bauernschuster et al., 2016^6^ | * | * | * | ** |  | * | * |  | ** | * | 10 |
| Baughman and Dickert-Conlin, 2009^7^ | * | * |  | ** |  | * | * |  | ** | * | 9 |
| Bonoli, 2008^8^ | * | * | * | ** |  | * | * |  | ** | * | 10 |
| Duvander et al., 2019^20^ | * | * | * | ** |  | * | * |  | ** | * | 10 |
| Einarsdóttir, 2023^22^ | * | * | * | ** |  | * | * |  | ** | * | 10 |
| Fukai, 2017^25^ | * | * |  | ** |  | * | * |  | ** | * | 9 |
| Gauthier and Hatzius, 1997^27^ | * | * |  | ** |  |  | * |  | ** | * | 8 |
| Harknett et al., 2014^30^ | * | * | * | ** |  | * | * |  | ** | * | 10 |
| Hart et al., 2022^31^ | * | * | * | ** |  | * | * |  | ** | * | 10 |
| Hong et al., 2016^32^ | * | * |  | ** |  | * | * |  | ** | * | 9 |
| Jeong et al., 2022^33^ | * | * | * | ** |  |  |  |  | ** | * | 8 |
| Lee and Lee, 2014^42^ | * | * | * | ** |  | * | * |  | ** | * | 10 |
| Luci-Greulich et a., 2013^45^ | * | * | * | ** |  | * | * |  | ** | * | 10 |
| Risse, 2006^52^ | * | * | * | ** |  | * | * |  | ** | * | 10 |
| Whittington, 1992^62^ | * | * | * | ** |  | * | * |  | ** | * | 10 |

Note: The cross-sectional studies used 10 criteria: the representativeness of the sample, sample size determination, non-response, ascertainment of exposure (maximum of five stars); comparability of the study on the basis of study design and analysis (maximum of two stars); and finally, the assessment of the outcome (maximum of three stars). All studies received a score on the basis of these nine criteria, ranging from 0 to 10. Studies were defined as high quality, score ≥ 6; moderate quality, score 4-5; or low quality, score 0-3.

Table S15: Quality assessment of intervention studies

| Study, year | Confounding bias | Detection Bias1 | Detection Bias2 | Selection Bias | Attrition Bias | Reporting Bias | Other Bias | Total Score |
| --- | --- | --- | --- | --- | --- | --- | --- | --- |
| Acs, 1996^3^ | Low | Low | Low | Low | Low | Low | Low | Low |
| Ang, 2015^4^ | Low | Low | Moderate | Low | Low | Low | Low | Low |
| Baizan and Arpino, 20163 | Low | Low | Moderate | Low | Low | Low | Low | Low |
| Bick A, 2016^7^ | Low | Low | Low | Low | Low | Low | Low | Low |
| Chen and Wei, 20228 | Low | Low | Low | Moderate | Low | Low | Low | Low |
| Chuard and Chuard-Keller, 2021^14^ | Low | Low | Low | Low | Low | Low | Low | Low |
| Cowan and Douds, 2022^15^ | Low | Low | Moderate | Low | Low | Low | Low | Low |
| Cygan-Rehm, 2015^16^ | Low | Low | Moderate | Low | Low | Low | Low | Low |
| Dahl and Loken, 2016^18^ | Low | Low | Low | Low | Low | Low | Low | Low |
| Einarsdóttir et al., 2012^21^ | Low | Low | Low | Low | Low | Low | Low | Low |
| Enache, 2013^23^ | Low | Low | Moderate | Low | Moderate | Low | Low | Low |
| Farréa and González, 2019^24^ | Low | Low | Low | Low | Low | Low | Low | Low |
| Gabos et al., 2009^26^ | Moderate | Low | Low | Moderate | Moderate | Moderate | Low | Moderate |
| Gohmann and Ohsfeldt, 1994^28^ | Moderate | Low | Low | Low | High | Moderate | Low | Moderate |
| González and Trommlerová, 2023^29^ | Low | Low | Low | Low | Low | Low | Low | Low |
| Jonsson, 2018^34^ | Low | Low | Low | Low | Low | Low | Low | Low |
| Kim and Luke, 2020^35^ | Low | Low | Low | Low | Low | Low | Low | Low |
| Kim and Parish, 2022^36^ | Low | Low | Low | Low | Low | Low | Low | Low |
| Kim, 2024^31^ | Low | Low | Moderate | Low | Low | Low | Low | Low |
| Kim, 2014^39^ | Low | Low | Moderate | Low | Low | Low | Low | Low |
| Lalive and Zweimülle, 2009^40^ | Low | Low | Low | Low | Low | Low | Low | Low |
| Malkov, 2018^46^ | Moderate | Low | Moderate | Low | Moderate | Moderate | Low | Moderate |
| Milligan, 2005^47^ | Low | Low | Low | Low | Low | Low | Low | Low |
| Neugart and Ohlsson, 2013^48^ | Low | Low | Moderate | Low | Low | Low | Low | Low |
| Parr and Guest, 2011^49^ | Low | Low | Low | Low | Low | Low | Low | Low |
| Raute, 2019^50^ | Moderate | Moderate | Low | Moderate | Moderate | Low | Low | Moderate |
| Ridao-Cano et al., 2005^51^ | Low | Low | Low | Low | Low | Low | Low | Low |
| Son 2017^56^ | Moderate | Moderate | Moderate | Moderate | Low | Moderate | Low | High |
| Speder et al. 2020^57^ | Moderate | Moderate | Low | Low | Moderate | Low | Low | Moderate |
| Thyrian et al., 2010^46^ | Low | Low | Low | Low | Low | Low | Low | Low |
| Yamaguchi S, 2019^47^ | Low | Low | Low | Moderate | Low | Low | Low | Low |
| Wesolowski et al., 2018^61^ | Moderate | Moderate | Low | Low | Moderate | Low | Low | Moderate |
| Winegarden and Bracy, 2019^63^ | Moderate | Moderate | Moderate | Low | Moderate | Low | Low | Moderate |

Note: Cochrane EPOC tools were used for assessing the risk of bias for quasi-experimental studies such as **c**ontrolled before and after, and interrupted time series ^2^. Risk of bias was evaluated through the following items: confounding bias, detection bias (only in non-experimental studies- two domains), selection bias, attrition bias, reporting bias, other bias.

(1) Confounding bias: Comparability of groups for quasi-experimental studies. For non-experimental studies, was the policy independent of other changes?

(2) Detection bias: Was the shape of the policy effect pre-specified? Was the policy unlikely to affect data collection?

(3) Selection bias: Sample representativeness.

(4) Attrition bias: Describe the completeness of outcome data for each main outcome, including attrition and exclusions from the analysis. - Reporting bias: State how the possibility of selective outcome reporting was examined by the review authors, and what was found.
(5) Other bias: State any important concerns about bias not addressed in the other domains in the tool.

**References**

1. Wells G SB, O'Connell D, Peterson J, Welch V, Losos M, et al. The Newcastle-Ottawa Scale (NOS) for assessing the quality of nonrandomised studies in meta-analyses. [*https://wwwohrica/programs/clinical_epidemiology/oxfordasp*](https://wwwohrica/programs/clinical_epidemiology/oxfordasp) 2015.

2. Effective Practice and Organization of Care (EPOC): EPOC resources for review authors. [*https://epoccochraneorg/resources/epoc-resources-review-authors*](https://epoccochraneorg/resources/epoc-resources-review-authors).

3. Acs G. The impact of welfare on young mothers' subsequent childbearing decisions. *Journal of Human Resources* 1996: 898-915.

4. Ang XL. The Effects of Cash Transfer Fertility Incentives and Parental Leave Benefits on Fertility and Labor Supply: Evidence from Two Natural Experiments. *JOURNAL OF FAMILY AND ECONOMIC ISSUES* 2015; **36**(2): 263-88.

5. Baizan P, Arpino B, Delclòs CE. The Effect of Gender Policies on Fertility: The Moderating Role of Education and Normative Context. *Eur J Popul* 2016; **32**(1): 1-30.

6. Bauernschuster S, Hener T, Rainer H. Children of a (policy) revolution: The introduction of universal child care and its effect on fertility. *Journal of the European Economic Association* 2016; **14**(4): 975-1005.

7. Baughman R, Dickert-Conlin S. The earned income tax credit and fertility. *JOURNAL OF POPULATION ECONOMICS* 2009; **22**(3): 537-63.

8. Bonoli G. The impact of social policy on fertility: evidence from Switzerland. *Journal of European social policy* 2008; **18**(1): 64-77.

9. Bick A. The quantitative role of child care for female labor force participation and fertility. *Journal of the European Economic Association* 2016; **14**(3): 639-68.

10. Bokun A. Cash transfers and fertility. *Demographic Research* 2024; **51**: 855-910.

11. Chen M, Lloyd CJ, Yip PSF. A new method of identifying target groups for pronatalist policy applied to Australia. *PLoS One* 2018; **13**(2): e0192007.

12. Cha W, Yun I, Nam C-M, Nam JY, Park E-C. Evaluation of Assisted Reproductive Technology Health Insurance Coverage for Multiple Pregnancies and Births in Korea. *JAMA network open* 2023; **6**(6): e2316696-e.

13. Chen H, Wei T, Wang H, et al. Association of China’s two-child policy with changes in number of births and birth defects rate, 2008–2017. *BMC Public Health* 2022; **22**(1): 434.

14. Chuard C, Chuard-Keller P. Baby bonus in Switzerland: Effects on fertility, newborn health, and birth-scheduling. *Health Econ* 2021; **30**(9): 2092-123.

15. Cowan SK, Douds KW. Examining the Effects of a Universal Cash Transfer on Fertility. *SOCIAL FORCES* 2022; **101**(2): 1003-30.

16. Cygan-Rehm K. Parental leave benefit and differential fertility responses: Evidence from a German reform. *Journal of Population Economics* 2016; **29**: 73-103.

17. Dinale D. The positive relationship between female employment and fertility rates: The role of family benefits expenditure and gender-role ideologies. *Journal of European Social Policy* 2024; **34**(4): 404-18.

18. Dahl GB, Loken KV, Mogstad M, Salvanes KV. What Is the Case for Paid Maternity Leave? *REVIEW OF ECONOMICS AND STATISTICS* 2016; **98**(4): 655-70.

19. Drago R, Sawyer K, Shreffler KM, Warren D, Wooden M. Did Australia's Baby Bonus Increase Fertility Intentions and Births? *POPULATION RESEARCH AND POLICY REVIEW* 2011; **30**(3): 381-97.

20. Duvander A-Z, Lappegård T, Andersen SN, Garðarsdóttir Ó, Neyer G, Viklund I. Parental leave policies and continued childbearing in Iceland, Norway, and Sweden. *Demographic Research* 2019; **40**: 1501-28.

21. Einarsdottir K, Langridge A, Hammond G, Gunnell AS, Haggar FA, Stanley FJ. The Australian Baby Bonus maternity payment and birth characteristics in Western Australia. *PloS one* 2012; **7**(11): e48885.

22. Einarsdóttir K. Changes in maximum parental leave payment in Iceland and total fertility rates. *Scand J Public Health* 2023; **51**(2): 197-203.

23. Enache C. Family and Childcare Support Public Expenditures and Short-Term Fertility Dynamics. *PANOECONOMICUS* 2013; **60**(3): 347-64.

24. Farré L, González L. Does paternity leave reduce fertility? *Journal of Public Economics* 2019; **172**: 52-66.

25. Fukai T. Childcare availability and fertility: Evidence from municipalities in Japan. *JOURNAL OF THE JAPANESE AND INTERNATIONAL ECONOMIES* 2017; **43**: 1-18.

26. Gábos A, Gál RI, Kézdi G. The effects of child-related benefits and pensions on fertility by birth order: A test on Hungarian data. *Population studies* 2009; **63**(3): 215-31.

27. Gauthier AH, Hatzius J. Family benefits and fertility: An econometric analysis. *Population studies* 1997; **51**(3): 295-306.

28. Gohmann SF, Ohsfeldt RL. THE DEPENDENT TAX-EXEMPTION, ABORTION AVAILABILITY, AND US FERTILITY RATES. *POPULATION RESEARCH AND POLICY REVIEW* 1994; **13**(4): 367-81.

29. González L, Trommlerová SK. Cash transfers and fertility: How the introduction and cancellation of a child benefit affected births and abortions. *Journal of Human Resources* 2023; **58**(3): 783-818.

30. Harknett K, Billari FC, Medalia C. Do family support environments influence fertility? Evidence from 20 European countries. *European Journal of Population* 2014; **30**: 1-33.

31. Hart RK, Andersen SN, Drange N. Effects of extended paternity leave on family dynamics. *JOURNAL OF MARRIAGE AND FAMILY* 2022; **84**(3): 814-39.

32. Hong SC, Kim YI, Lim JY, Yeo MY. Pro-natalist Cash Grants and Fertility: A Panel Analysis. *Korean Economic Review* 2016; **32**(2): 331-54.

33. Jeong K, Yoon J, Cho HJ, Kim S, Jang J. The relationship between changes in the korean fertility rate and policies to encourage fertility. *BMC PUBLIC HEALTH* 2022; **22**(1).

34. Jonsson AK. Family policies, childbearing, and economic crisis: The case of Iceland. *DEMOGRAPHIC RESEARCH* 2018; **39**: 561-92.

35. Kim J, Luke N. Lowest-Low Fertility in South Korea: Policy and Domestic Labor Supports and the Transition to Second Birth. *Social Forces* 2020; **99**(2): 700-31.

36. Kim EJ, Parish SL. Family-supportive workplace policies and benefits and fertility intentions in South Korea. *COMMUNITY WORK & FAMILY* 2022; **25**(4): 464-91.

37. Kim W. Baby bonus, fertility, and missing women. *Journal of Human Resources* 2024.

38. Kim S, Tertilt M, Yum M. Status externalities in education and low birth rates in Korea. *American Economic Review* 2024; **114**(6): 1576-611.

39. Kim YIA. Lifetime impact of cash transfer on fertility. *CANADIAN STUDIES IN POPULATION* 2014; **41**(1): 97-110.

40. Lalive R, Zweimüller J. How does parental leave affect fertility and return to work? Evidence from two natural experiments. *The Quarterly Journal of Economics* 2009; **124**(3): 1363-402.

41. Langridge AT, Nassar N, Li J, Jacoby P, Stanley FJ. The impact of monetary incentives on general fertility rates in Western Australia. *J Epidemiol Community Health* 2012; **66**(4): 296-301.

42. Lee GH, Lee SP. Childcare availability, fertility and female labor force participation in Japan. *Journal of the Japanese and International Economies* 2014; **32**: 71-85.

43. Li H-t, Xue M, Hellerstein S, et al. Association of China’s universal two child policy with changes in births and birth related health factors: national, descriptive comparative study. *Bmj* 2019; **366**.

44. Lin Y, Zhang B, Hu M, Yao Q, Jiang M, Zhu C. The effect of gradually lifting the two-child policy on demographic changes in China. *Health Policy and Planning* 2024; **39**(4): 363-71.

45. Luci-Greulich A, Thévenon O. The Impact of Family Policies on Fertility Trends in Developed Countries: L’influence des politiques familiales sur les tendances de la fécondité des pays développés. *European Journal of Population/Revue européenne de Démographie* 2013; **29**: 387-416.

46. Malkova O. Can Maternity Benefits Have Long-Term Effects on Childbearing? Evidence from Soviet Russia. *REVIEW OF ECONOMICS AND STATISTICS* 2018; **100**(4): 691-703.

47. Milligan K. Subsidizing the stork: New evidence on tax incentives and fertility. *Review of Economics and statistics* 2005; **87**(3): 539-55.

48. Neugart M, Ohlsson H. Economic incentives and the timing of births: evidence from the German parental benefit reform of 2007. *Journal of Population Economics* 2013; **26**: 87-108.

49. Parr N, Guest R. The contribution of increases in family benefits to Australia's early 21st-century fertility increase: An empirical analysis. *DEMOGRAPHIC RESEARCH* 2011; **25**: 215-44.

50. Raute A. Can financial incentives reduce the baby gap? Evidence from a reform in maternity leave benefits. *Journal of Public Economics* 2019; **169**: 203-22.

51. Ridao-Cano C, McNown R. The effect of tax-benefit policies on fertility and female labor force participation in the United States. *JOURNAL OF POLICY MODELING* 2005; **27**(9): 1083-96.

52. Risse L. Determinants of maternity leave provisions in Australia and the effects on fertility: An application of the Heckprobit selection model. Social Science Methodology Conference, University of Sydney; 2006; 2006.

53. Reich G. Determining the impact of the 2004 Australian Baby Bonus on fertility rates using a synthetic control analysis. *Australian Economic Papers* 2024; **63**: 23-32.

54. Sinclair S, Boymal J, De Silva A. A Re‐appraisal of the fertility response to the Australian baby bonus. *Economic Record* 2012; **88**: 78-87.

55. Soares CF, de Lima EEC. The Association between Conditional Cash Transfer Programmes and Cohort Fertility: Evidence from Brazil. *COMPARATIVE POPULATION STUDIES* 2021; **46**: 215-44.

56. Son YJ. Do childbirth grants increase the fertility rate? Policy impacts in South Korea. *Review of Economics of the Household* 2018; **16**(3): 713-35.

57. Spéder Z, Murinkó L, Oláh LS. Cash support vs. tax incentives: The differential impact of policy interventions on third births in contemporary Hungary. *Popul Stud (Camb)* 2020; **74**(1): 39-54.

58. Thyrian JR, Fendrich K, Lange A, Haas JP, Zygmunt M, Hoffmann W. Changing maternity leave policy: short-term effects on fertility rates and demographic variables in Germany. *Soc Sci Med* 2010; **71**(4): 672-6.

59. Yamaguchi S. Effects of parental leave policies on female career and fertility choices. *Quantitative Economics* 2019; **10**(3): 1195-232.

60. Yun I, Cha W, Nam C-M, Nam JY, Park E-C. The impact of assisted reproductive technology treatment coverage on marriage, pregnancy, and childbirth in women of childbearing age: an interrupted time-series analysis. *BMC Public Health* 2023; **23**(1): 1379.

61. Wesolowski K, Ferrarini T. Family policies and fertility: Examining the link between family policy institutions and fertility rates in 33 countries 1995-2011. *INTERNATIONAL JOURNAL OF SOCIOLOGY AND SOCIAL POLICY* 2018; **38**(11): 1057-70.

62. Whittington LA. Taxes and the family: the impact of the tax exemption for dependents on marital fertility. *Demography* 1992; **29**(2): 215-26.

63. Winegarden CR, Bracy PM. Demographic consequences of maternal-leave programs in industrial countries: evidence from fixed-effects models. *South Econ J* 1995; **61**(4): 1,020-35.
